# Endogenous rhythmic growth in oak trees is regulated by internal clocks rather than resource availability

**DOI:** 10.1093/jxb/erv408

**Published:** 2015-08-28

**Authors:** S. Herrmann, S. Recht, M. Boenn, L. Feldhahn, O. Angay, F. Fleischmann, M T. Tarkka, T.E.E. Grams, F. Buscot

**Affiliations:** ^1^Department of Soil Ecology, UFZ–Helmholtz Centre for Environmental Research, Theodor-Lieser-Str. 4, D-06120 Halle/Saale, Germany; ^2^Department of Community Ecology, UFZ–Helmholtz Centre for Environmental Research,Theodor-Lieser-Str. 4, D-06120 Halle/Saale, Germany; ^3^German Centre for Integrative Biodiversity Research (iDiv) Halle-Jena-Leipzig, Deutscher Platz 5e, D-04103 Leipzig, Germany; ^4^Section Pathology of Woody Plants, Technische Universität München, Hans-Carl-von-Carlowitz-Platz 2, D-85354 Freising, Germany; ^5^Ecophysiology of Plants, Technische Universität München, Hans-Carl-von-Carlowitz-Platz 2, D-85354 Freising, Germany

**Keywords:** Ectomycorrhiza, growth cessation, Piloderma croceum, *Quercus robur*, RNASeq, stable isotope labelling.

## Abstract

Common oak trees display endogenous rhythmic growth with alternating shoot and root flushes. To explore the mechanisms involved, microcuttings of the *Quercus robur* L. clone DF159 were used for ^13^C/^15^N labelling in combination with RNA sequencing (RNASeq) transcript profiling of shoots and roots. The effect of plant internal resource availability on the rhythmic growth of the cuttings was tested through inoculation with the ectomycorrhizal fungus *Piloderma croceum.* Shoot and root flushes were related to parallel shifts in above- and below-ground C and, to a lesser extent, N allocation. Increased plant internal resource availability by *P. croceum* inoculation with enhanced plant growth affected neither the rhythmic growth nor the associated resource allocation patterns. Two shifts in transcript abundance were identified during root and shoot growth cessation, and most concerned genes were down-regulated. Inoculation with *P. croceum* suppressed these transcript shifts in roots, but not in shoots. To identify core processes governing the rhythmic growth, functions [Gene Ontology (GO) terms] of the genes differentially expressed during the growth cessation in both leaves and roots of non-inoculated plants and leaves of *P*. *croceum*-inoculated plants were examined. Besides genes related to resource acquisition and cell development, which might reflect rather than trigger rhythmic growth, genes involved in signalling and/or regulated by the circadian clock were identified. The results indicate that rhythmic growth involves dramatic oscillations in plant metabolism and gene regulation between below- and above-ground parts. Ectomycorrhizal symbiosis may play a previously unsuspected role in smoothing these oscillations without modifying the rhythmic growth pattern.

## Highlight

Increased resource availability has no impact on endogenous rhythmic growth and related allocation shifts in oak trees; an internal clock regulates core genes during shoot and root growth cessation phases.

## Introduction

The growth of many trees is not continuous, but is interrupted by episodic rest phases (Kuehny *et al.*, 1997; McCown, 2000; Barthelemy and Caraglio, 2007). Pedunculate oak (*Quercus robur* L.), a major forest tree species in European forests, displays rhythmic growth with successive growth/rest phases of fixed periods of 18–22 d under constant culture conditions, which reflects the endogenic character of this trait (Klebs, 1913; Lavarenne, 1966; Champagnat *et al.*, 1986). Initial studies on rhythmic growth in young oak seedlings focused on the shoot and the role of shifts in carbohydrate concentration (Alaoui-Sossé *et al.*, 1994). Le Hir *et al.* (2005*a*) detected concomitance between shoot rhythmic growth and variations in sucrose synthase expression. They also related rhythmic shifts in sucrose concentration in apical tissues to alternating formation of normal and aborted leaves without lamina (Le Hir *et al.*, 2005*b*). To assess the effects of variations in downward soluble sugar export during shoot flushes, Willaume and Pages (2011) ablated source leaves and cotyledons of *Quercus pubescens* seedlings. This reduced the concentrations of non-structural carbohydrates in the seedlings’ root apices, and root growth. In young micropropagated apple trees, the growth of secondary roots rhythmically alternates with shoot flushes, in processes that Costes *et al.* (2006) hypothesized may be governed by oscillations in downward carbohydrate transfer, and possibly oscillations of nitrogen (N) metabolism. Similarly, *Q. robur* saplings older than 2 years display rhythmic development with alternating growth flushes of shoots and lateral roots (Reich *et al.*, 1980). Shifts in nitrogen allocation during rhythmic growth have been examined in stems of oak trees by Parmentier (1993) and in ornamental woody plants by Kuehny *et al.* (1997). However, the hypothesis of Costes *et al.* (2006) that balanced shoot and root rhythmic development is governed not only by carbon (C) but also by N partitioning has not yet been rigorously confirmed or refuted.

Interactions with mycorrhizal fungi should be considered in studies on the rhythmic growth of trees, because this ecologically obligatory symbiosis of roots not only enhances the acquisition of soil nutrients and stimulates photosynthesis of trees, but also changes their allocation among plant parts (Read and Perez-Moreno, 2003). In an optimized culture system, microcuttings of *Q. robur* (clone DF159) display full rhythmic growth with alternating shoot and root flushes (Herrmann *et al.*, 1998). In the presence of the ectomycorrhizal fungus *Piloderma croceum* J. Erikss. & Hjortst (strain F1598), the growth and photosynthesis of DF159 are highly stimulated, while photochemical stress is attenuated (Herrmann *et al.*, 2004). These strong effects are linked to differential gene expression (Frettinger *et al.*, 2007) and can be detected even in early stages of the association (Buscot and Herrmann, 2007).

Genomic and transcriptomic approaches are increasingly used to unravel episodic development in trees, but, to date, they have been largely applied to study mechanisms driven by exogenous factors such as seasonal variations (Olsen, 2010; Cooke *et al.*, 2012; Ueno *et al.*, 2013; Lesur *et al.*, 2015) or drought (Zawaski and Busov, 2014), rather than endogenous regulation. However, genomic resources on oaks are increasingly available (Ueno *et al.*, 2013; Lesur *et al.*, 2015). Notably, a transcriptome library of the DF159 clone containing >60 000 contigs (OakContigDF159.1) has been generated and already used for a broad transcriptomic study of its mycorrhizal association with *P. croceum* (Tarkka *et al.*, 2013).

In the study reported here, ^13^C and ^15^N stable isotope labelling were combined to follow resource allocation during rhythmic growth of the *Q. robur* clone DF159 with high-throughput Illumina transcript sequencing. Three hypotheses were tested. First, rhythmic growth with alternating shoot and root growth flushes is paralleled by shifts in the allocation of recently gained C and N toward the respective growing organs. Secondly, in spite of its growth-enhancing effect, the ectomycorrhizal fungus *P. croceum* impacts neither rhythmic growth nor relative resource allocation patterns between shoots and roots. Thirdly, rhythmic growth is regulated by leaves and roots via endogenous signalling rather than by resource availability.

## Materials and methods

### Experimental design and procedure

**Fig. 1.**
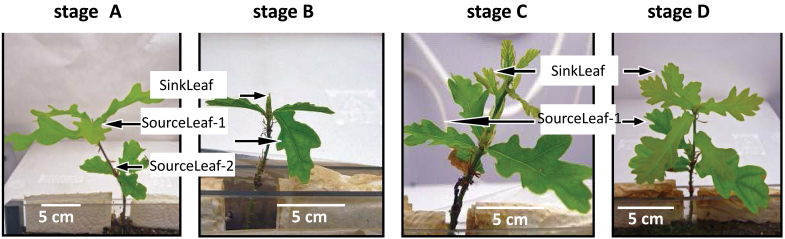
Developmental stages of *Quercus robur* L. microcuttings. Developmental stages A (bud rest), B (bud swelling), C (shoot elongation), and D (leaf expansion) showing source leaves (SourceLeaf-1 and SourceLeaf-2) and sink leaves (SinkLeaf).

Pedunculate oak (*Q. robur*) clone DF159 was micropropagated and rooted as previously described (Herrmann *et al.*, 2004; Herrmann and Buscot, 2008). Microcuttings were cultivated in a microcosm system derived from the one described by Herrmann *et al.* (1998), using 12×12cm^2^ Petri dishes and γ-radiation-sterilized soil (Tarkka *et al.*, 2013). Only rooted microcuttings in a phase shortly before a new shoot flushing were transferred into the microcosms. Half of the microcuttings were inoculated with the ectomycorrhizal fungus *P. croceum* (strain F1598) as described by Tarkka *et al.* (2013), and the others were used as non-inoculated controls. In order to restore the microbial community after γ-sterilization of the soil (Thuerig *et al.*, 2009), a 1/100 diluted suspension of native soil filtered through a 1.2 μm isoporous membrane (Millipore, Schwalbach, Germany) was added to the substrate 3 weeks after establishment of the microcosms. The development of the plants was monitored for 8 weeks in growth chambers providing 23±1 °C and long-day (16h light/8h dark) conditions with a photosynthetic photon flux density of ~180 μmol m^–2^ s^–1^ and 75% relative air humidity. Each microcosm was watered with sterilized tap water every 14 d using a sterile syringe. The developmental stage and numbers of established shoot flushes were recorded biweekly in a non-destructive manner. Four developmental stages were used to characterize each growth cycle: bud rest (A), bud swelling (B), shoot elongation (C), and leaf expansion (D) (Fig. 1). Stage B tightly correlates to maximal root elongation (Herrmann *et al.*, 1998) and, together with stage A, corresponds to a root flush. Stages C and D represent together a shoot flush. Under the experimental conditions employed, the first ectomycorrhizae formed during the fifth week, but few mycorrhizae were detected at harvest time (as expected since conditions, including relatively high air humidity and ample water supply, were selected that would minimize plant stress during the experiment).

C and N allocation patterns were assessed by stable isotope labelling. Three days before harvesting, 5ml of 0.02g l^–1 15^NH_4_^15^NO_3_ (98 atm% ^15^N, Sigma, Germany) were added to each root compartment under sterile conditions. For ^13^CO_2_ labelling, microcosms were transferred into a Plexiglas chamber 36h before harvest. During the night before labelling, the CO_2_ in the atmosphere was scrubbed with soda lime and replaced with 10 atm% ^13^CO_2_ (Eurisotop, Saarbrücken, Germany). During the following daytime period (16h), the CO_2_ concentration was adjusted to 400±2 μl l^–1^ (mean ±SD) with a ^13^C atm% of 7.9±0.3 (mean ±SD). Atmospheric CO_2_ was sampled every 4h. The isotopic composition of stable carbon isotopes of samples was assessed, using an isotope ratio mass spectrometer (Isoprime; Elementar, Hanau, Germany), within 3 d of sampling.

To compensate the low number and the low biomass of microcuttings at stage C which are inherent to the rhythmic growth (Supplementary Fig. S2A–D available at *JXB* online), additional plants at developmental stage C were used for analyses to provide a total of 65 control and 42 inoculated labelled microcuttings. Sets of plants were harvested in each of stages A, B, C, and D (Supplementary Fig. S2E). At harvest, each plant was divided into five fractions: principal roots, lateral roots, stems, and source and sink leaves (or buds for stage B) of the last two shoot flushes. All harvested leaves of plants at bud rest stage A were source leaves (Fig. 1) of the terminal or subterminal flushes (SourceLeaf-1 and SourceLeaf-2, respectively). In developmental stages B, C, and D, SourceLeaf-1 of the subterminal flush and sink leaves including buds (SinkLeaf) were harvested (Fig. 1). After FW determination, all plant fractions were immediately submerged in liquid nitrogen and stored at –80 °C.

### Isotope ratio mass spectrometry analysis, and quantification of non-structural carbohydrates

The DW of each plant fraction was measured after freeze-drying for 24h (using a Beta1-8 system, Martin Christ, Osterode am Harz, Germany) to establish FW to DW ratios. ^13^C/^12^C and ^15^N/^14^N isotope ratios (expressed as excess levels of ^13^C and ^15^N, i.e. the difference between measured and natural ratios) were determined for all organ fractions of 2–3 plants per treatment and developmental stage. The isotope ratios of 2mg portions of the freeze-dried and finely milled plant material were determined using a GVI-Isoprime isotope ratio mass spectrometer (Elementar) coupled to an EA3000 element analyzer (Euro Vector, Milan, Italy). Repeated measurements of a laboratory working standard gave a precision of δ^13^C <0.1 ‰ (SD, *n*=10). ^13^C and ^15^N excess levels were calculated for both individual plant organs and entire plants using unlabelled microcuttings as controls. Soluble sugar and starch contents were analysed according to Angay *et al.* (2014).

### Gene expression profiling

Transcriptome analyses focused on the most distal fully developed leaves and lateral roots (LRs). The frozen samples were split into 50mg portions for leaves and 100mg for LRs. SourceLeaf-1 at stages A, B, C, and D, SinkLeaf at stages C and D, and LR samples were pooled to obtain three biological replicates of these organs under both treatments (except LR samples of inoculated plants at stage C for which only two replicates were available), giving 57 samples for RNA analysis in total. RNA was extracted using a MasterPure Plant RNA Purification Kit (Epicentre, Germany) and its quality was verified according to Tarkka *et al.* (2013). It was then sequenced at the Beijing Genomics Institute (China), using the Illumina HiSeq 2000 platform. For library preparation, a minimum of 0.6 μg of total RNA per sample was used after optimization by standard Illumina procedures to generate 100bp sequences from paired-end libraries (average insert size 198bp). The numbers of paired-end reads ranged from 13 001 295 to 15 617 400 (Supplementary Table S1 at *JXB* online). The Illumina reads have been deposited to the short reads archive with the accession number PRJNA268569. Reads were pre-processed and transcript abundance quantified according to Tarkka *et al.* (2013). The OakContigDF159.1 reference library, Gene Ontology (GO) annotations, and best blast hits of each contig have been deposited at www.trophinoak.de. Homologues for oak contigs were determined by BLASTX search against the TAIR database. To validate the differential contig expression results, levels of eight contigs that were differentially expressed in leaves between stages D and A, and of five that were differentially expressed in LRs between stages B and C were quantified by quantitative real-time PCR (qRT-PCR), using primers selected and tested according to Tarkka *et al.* (2013) (Supplementary Fig. S1; Table S2). Pairwise comparisons were used to assess differences in contig expression profiles of leaves (Leaf) and lateral roots (LRs) between successive developmental stages [i.e. from D to A (_Dto_A), A to B (_Ato_B), B to C (_Bto_C), and C to D (_Cto_D) (Supplementary Table S1). SourceLeaf-1 was used in all comparisons of leaf profiles, except in _Dto_A, in which SinkLeaf at stage D was compared with SourceLeaf-1 at stage A (Fig. 1). Differentially expressed contigs (DECs) which occurred in pairwise comparisons between successive developmental stages and were common to both control and *P. croceum*-inoculated plants were termed intersection Cont&Pi genes. Further intersections containing DECs common to leaves and roots at comparable steps of their development were subsequently inspected, and analyses were focused on intersections with the highest number of common DECs (Supplementary Table S3).

### Statistical analyses

Effects of developmental stages and inoculation with *P. croceum* on measured DW, and isotope ratios were analysed with two-way ANOVA using R software (R core group, http://www.r-project.org/). Under the experimental conditions used, shoot and root biomass did not significantly differ between plants that had displayed two or three flushes at any final developmental stage, except for control plants at stages 2D and 3D (*P*<0.05). Therefore, for the analyses, samples of plants harvested in developmental stages A, B, C, or D were pooled, regardless of the number of shoot flushes they had displayed (Supplementary Fig. S2E at *JXB* online). Differences between stages are given for shoot and root parts and whole plants using a Tukey test at a *P*-value <0.05. The same test was applied for modifications between successive stages (indicated by log2 ratios) of ^13^C and ^15^N excess, and non-structural carbohydrates within leaves and lateral roots.

Differences in contig expression were determined using the edgeR function (Robinson *et al*., 2010) of the Bioconductor package in R (Gentleman *et al.*, 2004), and were considered to be significant when Benjamini–Hochberg-adjusted *P*-values were <0.01 (Supplementary Tables S1, S3 at *JXB* online). To assess the statistical significance of enrichment of GO terms among the sets of DECs, GOseq was used (Young *et al.*, 2010), which is designed to overcome the length bias inherent in RNA sequence data. GO terms were considered to be significantly enriched when *P*-values were <0.05. The same threshold was used to assess the significance of GO term enrichment for the intersections of lists of DECs from pairwise comparisons (Supplementary Tables S4, S5).

## Results

### Expression of the endogenous rhythmic growth demonstrates that the fungus does not accelerate growth rhythm

**Fig. 2.**
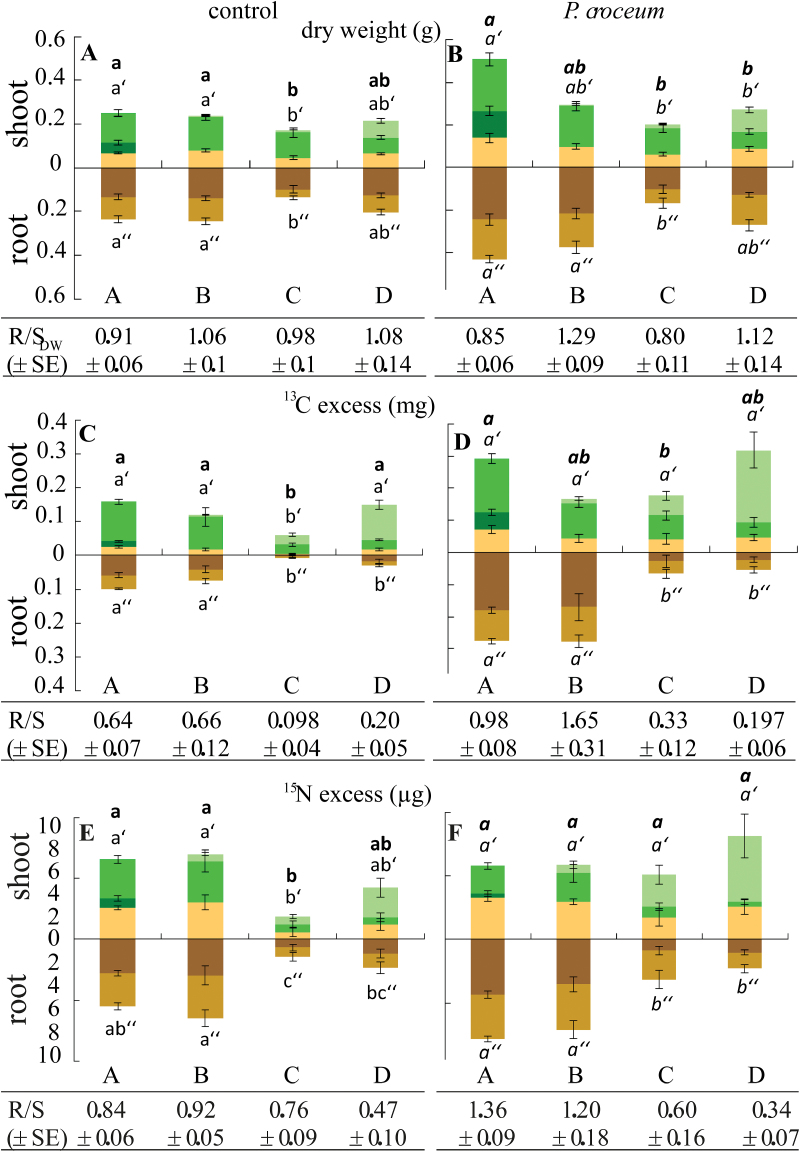
Biomass and both ^13^C and ^15^N excess of oak plant fractions at the four developmental stages. Measurements performed for harvested plant fractions [SinkLeaf (light green), SourceLeaf-1 (med green), and SourceLeaf-2 (dark green), stem (ochre), principal roots (dark brown), and lateral roots (light brown)] at the four developmental stages (A, B, C, and D): DWs in (A) control and (B) *Piloderma croceum*-inoculated oak microcuttings; ^13^C excess levels of (C) control and (D) *P. croceum*-inoculated plants; and ^15^N excess levels of (E) control and (F) *P. croceum*-inoculated plants. Root to shoot ratios (R/S_DW,_ R/S_13C_, and R/S_15N_) determined for plants at each of the four stages under each treatment are shown (means ±SE). Significant differences between stages are indicated with different letters for whole control plants (**a, b**), shoots (a’, b’), and roots (a’’, b’’) and, similarly, but in italics, for *P. croceum*-inoculated plants; Tukey test at *P*<0.05.

Although all plants were in bud swelling stage B at the beginning of the experiment, they did not grow synchronously. After just 2 weeks, the four developmental stages A (bud rest), B (bud swelling), C (shoot elongation), and D (leaf expansion) (Fig. 1) were represented and ranged from 1B to 2D (Supplementary Fig. S2A, B at *JXB* online). After 8 weeks, the fewest microcuttings were observed in shoot elongation stage C due to the transience of this stage (Supplementary Fig. S2A, B). Periods of the growth cycles (GCs) were calculated based on dates for the end of stage B, just before the start of stage C in consecutive cycles. This yielded similar periods of 37.19±3.71 d and 34.3±4.56 d for GC2 from 1B to 2B, and 28.29±3.8 d and 27.6±2.10 d for GC3 from 2B to 3B, in control and *P. croceum*-inoculated microcuttings, respectively (Supplementary Fig. S2A, B). The highly similar distribution of plants at different developmental stages in control and *P. croceum*-treated plants shows that the fungus did not accelerate the growth rhythm. The two distributions were strongly correlated, with a Spearman’s correlation coefficient of 0.962 (*P=*3.838e-08). The plants harvested at shoot elongation stage C had the lowest biomass, and those harvested at stages A and B, during root flushing, had the highest biomass (Fig. 2A; Table 1). Inoculation with *P. croceum* enhanced the plant biomass at every stage, without modifying the impact of the developmental stage, except for marginal stage×*P. croceum* interaction effects on the biomass of source leaves and principal roots (Fig. 2B; Table 1). Finally, the fungus affected neither the number nor the duration of the GCs during rhythmic growth.

**Table 1.** Results of the two-way ANOVA Significance levels (*P*-values) for the main effects of developmental stage (stage), inoculation by *Piloderma croceum,* and their interaction (stage×*P. croceum*) on DW, ^13^C excess, and ^15^N excess, in the different fractions of *Q. robur* microcuttings.

	Stage	*P. croceum*	Stage×*P. croceum*
**DW (g**)			
Total plant	***	**	NS
Total shoot	**	*	NS
Total roots	***	***	NS
Sink leaves (SiLeaf)	***	NS	NS
Source leaves (SoLeaf-1)	***	*	(*)
Stem	*	***	NS
Principal roots (PRS)	***	**	(*)
Lateral roots (LRS)	**	***	NS
R/S_DW_	NS	NS	NS
**Excess ^13^C (mg**)			
Total plant	***	***	NS
Total shoot	***	***	NS
Total roots	***	***	*
Sink leaves (SiLeaf)	***	*	(*)
Source leaves (SoLeaf-1)	***	**	NS
Stem	*	***	NS
Principal roots (PRs)	***	***	**
Lateral roots (LRs)	***	***	(*)
R/S_13C_	***	***	(*)
**Excess ^15^N (μg**)			
Total plant	***	(*)	NS
Total shoot	*	n.s.	(*)
Total roots	***	(*)	NS
Sink leaves (SiLeaf)	**	(*)	NS
Source leaves (SoLeaf-1)	***	NS	NS
Stem	**	(*)	NS
Principal roots (PRs)	***	NS	NS
Lateral roots (LRs)	***	(*)	NS
R/S_15N_	NS	NS	NS

NS, non-significant; (*)*P*<0.05; *P<0.01; **P<0.001; ***P<0.0001.

### ^13^C and ^15^N excess and non-structural carbohydrates vary during rhythmic growth; *P. croceum* increases ^13^C excess over the whole growth cycle without modifying its profile

In control plants, total ^13^C and ^15^N excess levels were significantly dependent on the developmental stage reached at harvest (Fig. 2C, E; Table 1). They were both lowest in plants at shoot elongation stage C, highest in plants at root flush stages A and B, and showed similar patterns to DW profiles (Fig. 2A). *Piloderma croceum* inoculation induced increases in total ^13^C excess in all stages, but did not greatly modify the total ^13^C excess profile observed across the four stages (stage×*P. croceum* in Table 1). Significant stage×*P. croceum* interaction effects were observed on ^13^C excess only for principal roots (*P*<0.001) and to a lower extent in total roots (*P*<0.01) (Fig. 2D; Table 1). ^15^N excess was only marginally affected by the fungus (Fig. 2F; Table 1). Pairwise comparisons of log2 ratios of ^13^C and ^15^N excess confirmed that there were significant shifts in ^13^C and ^15^N excess during transitions B to C (_Bto_C) in leaves and LRs (Table 2). After the transition B to C, the starch content decreased in the source leaves and LRs. In the presence of *P. croceum*, starch utilization to support the next shoot flush remained unaffected (Table 2).

**Table 2.** *Log2 ratios based on pairwise comparisons of* ^*13*^ *C excess,* ^*15*^ *N excess, and soluble sugar and starch contents in leaves (Leaf) and lateral roots (LRs) between successive developmental stages A (bud rest), B (bud swelling), C (shoot elongation), and D (leaf expansion) of control (Cont) and* P. croceum*-inoculated (Pi) oak microcuttings* Positive/negative log2 ratios indicate increasing/decreasing values between the initial and final stage of the pairwise comparison.

	Excess ^13^C				Excess ^15^N				Soluble sugar				Starch			
	Cont_Leaf		Cont_LR		Cont_ Leaf		Cont_LR		Cont_Leaf		Cont_LR		Cont_Leaf		Cont_LR	
_Dto_A	0.18	NS	1.79	**	0.40	NS	1.25	NS	1.85	NS	1.61	*	2.31	NS	3.72	(*)
_Ato_B	–0.28	NS	–0.24	NS	0.07	NS	0.36	NS	0.49	NS	–0.65	NS	1.11	*	0.62	NS
_Bto_C	–1.83	*	–3.49	***	–2.44	*	–2.22	**	0.08	NS	–2.54	*	–2.35	***	–3.82	***
_Cto_D	0.05	NS	1.95	NS	–0.13	NS	0.61	NS	–0.46	NS	1.58	NS	–1.07	NS	–0.53	NS
	**Pi_Leaf**		**Pi_LR**		**Pi_ Leaf**		**Pi_LR**		**Pi_Leaf**		**Pi_LR**		**Pi_Leaf**		**Pi_LR**	
_Dto_A	–0.42	NS	1.64	(*)	–1.24	NS	1.44	(*)	1.31	*	1.69	*	2.38	NS	3.95	***
_Ato_B	–0.63	NS	0.17	NS	0.02	NS	0.04	NS	0.24	NS	–0.15	NS	0.68	NS	0.02	NS
_Bto_C	–0.55	NS	–1.45	*	–1.41	NS	–0.66	NS	–0.19	NS	–1.86	*	–1.87	*	–3.32	**
_Cto_D	–0.59	NS	–0.36	NS	–1.25	NS	–0.82	NS	–0.68	NS	0.32	NS	–1.19	NS	–0.64	NS

NS, non-significant; (*)*P*<0.05; *P<0.01; **P<0.001; ***P<0.0001.

### Relative allocation of ^13^C and ^15^N between the shoot and root compartments reveals no impact of *P. croceum* on C allocation

Relative C allocation to the shoot and root compartments, expressed as R/S ratios of ^13^C excess, significantly changed with the developmental stage and shifted toward the roots during root flushing stages A and B and toward the shoots during shoot flushing stages C and D (Fig. 2C; Table 1). No such significant shifts were detected in N allocation (Fig. 2E; Table 1). In the presence of *P. croceum*, the pattern of predominant C allocation towards the roots during root flushing and the shoots during shoot flushing was maintained, and the interactive effect between developmental stage and *P. croceum* inoculation (stage×*P. croceum*) on R/S_13C_ was only marginally significant (Table 1), indicating that *P. croceum* did not strongly affect C partitioning within the plants. The fungus had no significant effect on the N partitioning across the four developmental stages (Fig. 2F; Table 1).

### Differential contig expression common to leaves and roots and to *P. croceum*-inoculated plants may reveal core genes governing rhythmic growth

In control plants, two peaks in numbers of DECs were detected in the pairwise comparison of leaves (Cont_Leaf) in the stage transition _Dto_A (3138) and of LRs (Cont_LR) in the stage transition _Bto_C (4353) during shoot growth cessation (SGC) and root growth cessation (RGC), respectively (Supplementary Table S6 at *JXB* online). In both cases, roughly a third of the contigs were up-regulated and two-thirds down-regulated. After inoculation with *P. croceum*, differential expression was strongly reduced: only 1144 DECs were detected during SGC and 37 DECs during RGC (Supplementary Table S6). Comparison of log2 fold changes (log2 FCs) of DECs in LRs with log2 ratios in ^13^C and ^15^N excess, and contents of non-structural carbohydrates (Table 2) revealed the coinciding drop in log2 FCs and log2 ratios in the shoot elongation stage C.

**Fig. 3.**
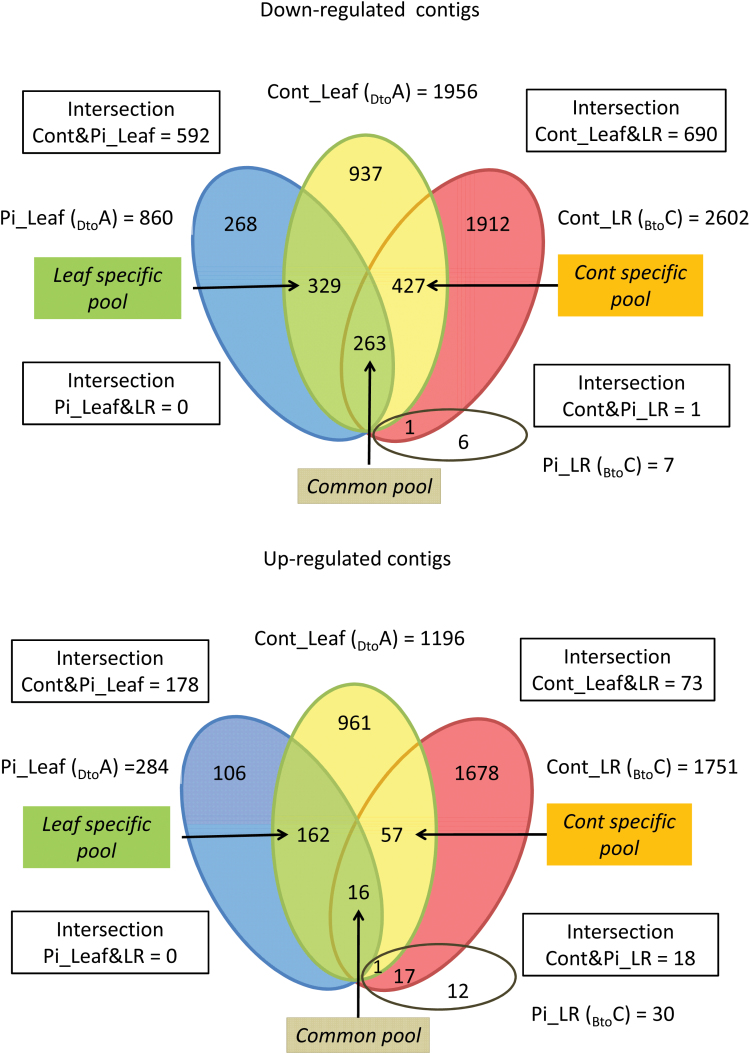
Venn diagrams based on up- and down-regulated contigs in leaves (Leaf) and lateral roots (LRs) within the Control (Cont) and *P. croceum*-inoculated (Pi) oak microcuttings. Differential expression of contigs was calculated in leaves (Leaf) by pairwise comparisons from stages D to A (_Dto_A) at shoot growth cessation and in LRs from stages B to C (_Bto_C) at root growth cessation. The blue ellipse marks 284 DECs in inoculated plants at leaf growth cessation, the yellow ellipse 1196 DECs in control plants at leaf growth cessation, and the red ellipse 1752 DECs of control plants during root growth cessation. Green colour indicates the ‘Leaf specific pool’ with DECs exclusively present at leaf growth cessation in control and inoculated plants, orange the ‘Cont specific pool’ with DECs exclusively present in control plants at shoot and root growth cessation, and beige the ‘Common pool’ with DECs belonging to control and inoculated plants at shoot and root growth cessation.

The results presented above showed that *P. croceum* treatment did not affect the period of rhythmic growth. Thus, common DECs in pairwise comparisons between stages were identified and assessed as core genes. Partly following Venn diagram terminology, the core genes differentially expressed in both control (Cont) and *P. croceum*-inoculated (Pi) plants were termed intersection Cont&Pi genes. It was expected that these intersection sets would include regulators of endogenous rhythmic growth. Furthermore, as the numbers of DECs were highest in alternating growth phases, concomitantly with growth cessation of the corresponding plant organs, further intersections containing core genes common to SGC and RGC in control and Pi plants were inspected. In analysis of DECs detected in both control and Pi plants, a maximal number of 770 common DECs were found in leaves at SGC and a maximum of just 19 DECs in LRs at RGC (Fig. 3). In analysis of the DECs common to leaves and roots during growth cessation, 763 DECs were found for control plants, but no common DECs after *P. croceum* inoculation (Fig. 3).

### GO terms involved in C and N metabolism are specifically enriched in control leaves and roots during growth cessation

Results of the GO term analyses of the DECs in the pairwise comparisons of successive stages are given in Supplementary Table S4 at *JXB* online. The analyses were focused on the transitions _Dto_A for leaves of control and Pi plants (Cont_Leaf and Pi_Leaf) and _Bto_C for LRs in control plants (Cont_LR). Both transitions _Dto_A and _Bto_C correspond to SGC and RGC, respectively. The comparisons Cont_Leaf revealed that GO terms related to photosynthesis (‘response to carbon dioxide’, ‘regulation of photosynthesis’, ‘photosynthetic acclimation’, and ‘glucose-6-phosphate transport’) were specifically enriched in up-regulated contigs (Supplementary Table S7). No enrichment of genes involved in N metabolism was found in Cont_Leaf. In Cont_LR, numerous GO terms associated with the catabolism, metabolism, and biosynthesis of several amino acids were specifically enriched among down- and up-regulated contigs (Supplementary Table S7). A down-regulation at the transcriptome level is consistent with both the reduced log2 ratio in ^15^N excess in LRs, and distinct reduction of ^15^N excess in the whole root system during the RGC (Fig. 2E). GO terms involved in C metabolism including ‘carbon utilization’ and ‘trehalose biosynthetic process’ were enriched, with 10 up-regulated trehalose 6-phosphate synthase contigs in Cont_LR. The apparent enhancement of local C utilization in roots probably counterbalances the reduction in carbohydrate transport to LRs during RGC since the GO term ‘UDP-glucose transport’ was enriched in down-regulated contigs.

### GO terms particularly enriched during root and shoot growth cessation

Further analyses focused on the two intersections with the highest numbers of common DECs, Cont&Pi_Leaf and Cont_Leaf&LR, for which three pools of DECs were considered (Fig. 3). A first pool Cont&Pi_Leaf–Cont_Leaf&LR termed ‘Leaf specific pool’ consists of contigs that were differentially expressed only in leaves during SGC and were not influenced by *P. croceum*. A second pool common to the two intersections Cont&Pi_Leaf–Cont_Leaf&LR termed ‘Common pool’ consists of contigs that were differentially expressed only in leaves and LRs during SGC and RGC whose expression was modified by *P. croceum* in roots but not in leaves. A third pool Cont_Leaf&LR–ContPi_Leaf termed ‘Cont specific pool’ consists of contigs that were differentially expressed in leaves and LRs during SGC and RGC in control plants but which are influenced by *P. croceum* in both leaves and roots.

Results of the GO term analyses of DECs in the focal intersections and pools are presented in Supplementary Table S7 at *JXB* online. In the ‘Common pool’ of DECs, there was strong enrichment in down-regulated contigs of GO terms associated with C metabolism (e.g. ‘metabolic processes’, ‘UDP-glucose 6-dehydrogenase activity’ and ‘UDP-glucuronate decarboxylase activity’) and cell development (e.g. ‘regulation of meristem growth’, ‘plant-type cell wall organization’, microtubule-based movement’, and ‘microtubule nucleation’). In the ‘Common pool’, GO terms ‘gibberellic acid-mediated signalling pathway’ and ‘auxin polar transport’ were enriched in down-regulated contigs. GO terms enriched in the Cont&Pi_Leaf intersection corroborated the previously outlined leaf specificity of DECs involved in C metabolism, with enrichment in up-regulated contigs for GO terms ‘sucrose transport’ and ‘starch metabolic process’ and in down-regulated contigs for the GO terms ‘sucrose metabolic process’ and ‘sucrose catabolic process’. Further enrichment of GO terms with contigs related to auxin, cytokinin, and salicylic acid were also specifically identified in the Cont&Pi_Leaf set. Finally, GO terms associated with flowering, photoperiodism, cold acclimation, or circadian regulation were enriched in DECs of the Cont_Leaf&LR set, and genes involved in the red light signalling pathway were enriched in the Cont&Pi_Leaf intersection.

### Differentially expressed core genes are mainly down-regulated during growth cessation

Based on the results of the GO term analysis, analyses of core genes were restricted to DECs involved in C and N metabolism, cell development, hormonal regulation, and/or presumably involved in flowering, photoperiodism, or the circadian clock. In total, 756 down- and 235 up-regulated contigs were found for the three pools, confirming the dominance of down-regulation of core genes associated with growth cessation during rhythmic development (Fig. 3). Most striking was the low number of 16 up-regulated contigs in the ‘Common pool’ compared with the 263 down-regulated contigs (Fig. 3). Furthermore, only 73 contigs were up-regulated in control plants during SGC and RGC (Cont_Leaf&LR intersection). Genes with differential expression restricted to leaves were found in the ‘Leaf specific pool’ demonstrating that *P. croceum* had no effect on differential expression of these leaf genes.

#### Regulation of C and N metabolic genes

**Fig. 4.**
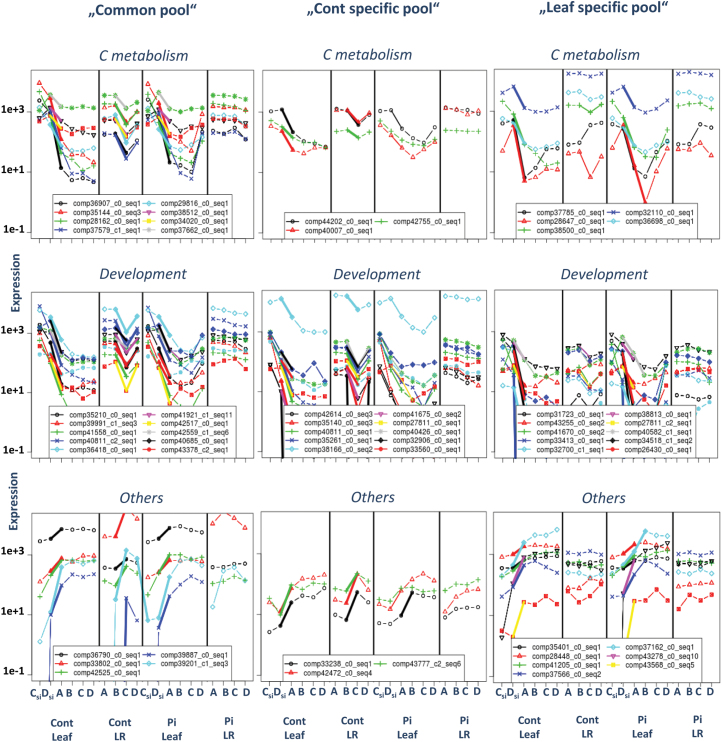
Profiles of transcript abundance of selected differentially expressed contigs (DECs) within ‘Common pool’, ‘Cont specific pool’, and ‘Leaf specific pool’ resulting from the intersections Cont&Pi_Leaf and Cont_Leaf&LR at growth cessation: in the ‘Common pool’, DECs are common to LRs and leaves of controls and leaves of inoculated oak microcuttings; in the ‘Leaf specific pool’ DECs are common to control and inoculated leaves; and in the ‘Cont specific pool’ DECs are common to leaves and LRs of control plants. The transcript abundance is given for leaves (Leaf) and roots (LR) of control (Cont) and *P. croceum*-inoculated (Pi) plants at the four developmental stages A (bud rest), B (bud swelling), C (shoot elongation), and D (leaf expansion). Except for ‘si’ (indicating SinkLeaves), all leaves were SourceLeaf-1. Significant differential expression of transcripts between stages at *P*<0.01 is given in bold lines.

DECs encoding C metabolism genes were mostly represented in the two pools ‘Common pool’ and ‘Leaf specific pool’ (Supplementary Table S8 at *JXB* online). In the ‘Common pool’, all C metabolic contigs were down-regulated (Supplementary Table S8). Variations of transcript abundance over the four developmental stages for down-regulated contigs encoding C metabolic genes in the ‘Cont specific pool’ revealed a non-significant drop in transcript abundance in inoculated leaves, reflecting the smoothing effect of *P. croceum* on the differential expression (Fig. 4). Contigs of the ‘Leaf specific pool’ were strongly differentially expressed in leaves during SGC (comp37785_c0_seq1, comp32110_c0_seq1, and comp36698_c0_seq1 in Table 3; Fig. 4). Both acid invertase and sucrose synthase were found to be down-regulated in the ‘Leaf specific pool’ (Table 3). Contigs encoding genes involved in N transport were slightly differentially expressed and less represented in the ‘Cont specific pool’ (Supplementary Table S8).

**Table 3.** *Selection of differential expressed contigs (DECs) (for a more extensive list see* Supplementary Table S8 *at* JXB *online) encoding genes with high log2 FC and/or high similarities (E-values) to* A. thaliana *genes (Blastx in TAIR) represented in the three pools resulting from the intersections Cont&Pi_Leaf and Cont_Leaf&LR at shoot growth cessation (SGC) in leaves (Leaf) and root growth cessation (RGC) in lateral roots (LRs) of control (Cont) and P. croceum inoculated (Pi) plants* In the ‘Common pool’ DECs are common to LRs and leaves of controls and leaves of inoculated oak microcuttings, in the ‘Leaf specific pool’ DECs are common to control and inoculated leaves, in the ‘Cont specific pool’ DECS are common to leaves and LRs of control plants. Log2 FCs of the Benjamin–Hochberg-adjusted *P*-values (*P*-adj<0.01) are given in bold (blue fields), for down-regulated genes and in italics (red fields) for up-regulated genes in the transition D to A (LeafDtoA) during SGC and in the transition B to C (LRBtoC) during RGC. Predicted functions originate from Blast2GO annotation of the OakContigDF159.1 reference library (Tarkka *et al.*, 2013).

Predicted functions	OakDF159 Contig name	Common pool	Cont specific pool	Leaf specific pool	Cont Leaf_Dto_A	Cont LR_Bto_C	Pi Leaf_Dto_A	Gene in *A. thaliana*	Alignment E-value	Encoded proteins in *A. thaliana*
**C metabolism**										
Endo-beta-glucanase	comp36907_c0_seq1	x			**–6.05**	**–2.11**	**–4.98**	AT1G70710	0.0	GH9B1. CEL1.
*o*-Glycosyl hydrolases family 17	comp41738_c0_seq1	x			**–1.70**	**–2.45**	**–1.76**	AT4G14080	1e-153	MEE48
beta-Glucanase	comp44202_c0_seq1		x		**–2.46**	**–1.56**		AT3G07320	0.0	GH17
Glucan endo-beta-glucosidase	comp40819_c0_seq1		x		*2.91*	*1.70*		AT5G42720	5e-73	GH17
Acid invertase	comp37785_c0_seq1			x	**–6.22**		**–4.32**	AT1G12240	0.0	ATBETAFRUCT4. V
Sucrose synthase sus1	comp32110_c0_seq1			x	**–2.32**		**–2.24**	AT5G20830	0.0	SUS1.
*o*-Glycosyl hydrolases family 17	comp36698_c0_seq1			x	**–2.29**		**–2.29**	AT5G55180	1e-178	GH17
Nodulin 3 family protein	comp40679_c0_seq1			x	*2.03*		*3.03*	AT5G23660	2e-47	MTN3. SWEET12.
**N metabolism**										
Ammonium transporter amt2	comp38427_c0_seq1		x		*1.77*	*1.29*		AT2G38290	0.0	ATAMT2. AMT2;1.
Probable peptide nitrate transporter	comp43246_c1_seq1		x		*1.84*	*2.27*		AT1G27080	7e-81	NRT1.6
**Gibberellins**										
Gibberellin-regulated protein	comp29599_c0_seq1	x			**–6.58**	**–3.49**	**–7.59**	AT5G15230	9e-32	GASA4 | GAST1
Gibberellin 20-oxidase	comp7575_c0_seq1			x	**–6.86**		**–6.43**	AT5G51810	1e-44	GA20OX2.
**Auxins**										
Auxin influx carrier component	comp38228_c2_seq1		x		**–8.59**	**–2.13**		AT2G21050	0.0	LAX2
MtN21 nodulin family transporter	comp37627_c0_seq2			x	**–10.38**		**–9.53**	AT3G53210	1e-100	UMAMIT6
Cytokinins										
Purine permease	comp43663_c0_seq1			x	*1.62*		*1.37*	AT1G28230	3e-68	PUP1. ATPUP1
**Cell development**										
Leucine-rich repeat receptor-like ki pxl2	comp42644_c0_seq2	x			**–5.05**	**–3.90**	**–6.42**	AT5G61480	6e-95	PXY
Leucine-rich repeat receptor-like kinase	comp39844_c0_seq2	x			**–2.73**	**–1.39**	**–2.26**	AT4G18640	1e-174	MRH1
Receptor protein kinase clavata1	comp38911_c1_seq1	x			**–2.47**	**–0.98**	**–1.91**	AT1G75820	1e-172	CLV1. FAS3. FLO5
Leucine-rich repeat receptor-like kinase	comp42887_c1_seq1	x			**–1.04**	**–1.22**	**–1.19**	AT1G28440	0.0	HSL1 | HAESA-like 1
Cyclin a2	comp41558_c0_seq1	x			**–3.43**	**–2.40**	**–3.17**	AT1G15570	1e-119	CYCA2;3 | CYCLIN A2;3
Cyclin d3	comp36768_c4_seq1	x			**–3.25**	**–1.21**	**–2.57**	AT5G67260	5e-82	CYCD3;2 | CYCLIN D3;2
**Transcription factors**										
Gras family transcription factor	comp39403_c0_seq1	x			**–3.15**	**–1.75**	**–2.54**	AT1G63100	0.0	GRAS TF
Platz transcription factor domain	comp43527_c1_seq3	x			*1.48*	*2.73*	*1.66*	AT1G21000	3e-97	PLATZ TF
Transcription factor bhlh62-like	comp34576_c0_seq1		x		**–2.01**	**–1.15**		AT1G10120	6e-44	CIB2
Transcription factor myb48	comp33951_c0_seq4		x		*1.68*	*1.25*		AT3G46130	4e-53	MYB48
Wrky transcription	comp22770_c0_seq1		x		*3.22*	*3.85*		AT5G13080	1e-41	WRKY75. ATWRKY75.
Transcription factor bhlh63-like	comp29996_c0_seq2			x	**–2.89**		**–2.33**	AT4G34530	9e-51	CIB1
Zinc finger	comp35351_c0_seq1			x	**–10.22**		**–10.15**	AT5G33370	1e-145	GDSL-like lipase
**Other genes**										
Quasimodo1-like protein	comp42130_c0_seq1	x			**–1.17**	**–1.07**	**–1.07**	AT3G25140	0.0	GAUT8. QUA1.
Multidrug resistance	comp42525_c0_seq1	x			*1.69*	*2.12*	*2.00*	AT5G65380	0.0	MATE efflux family
Protein proliferation	comp39887_c0_seq1	x			*3.25*	*8.21*	*3.47*	AT4G02060	0.0	PRL. MCM7.
E3 ubiquitin-protein ligase xbat31-like	comp39201_c1_seq3	x			*5.00*	*5.44*	*4.49*	AT2G28840	1e-154	XBAT31 | XB3 orthologue 1
Transducin wd-40 repeat-containing	comp43499_c0_seq2		x		**–8.81**	**–6.68**		AT3G06880	1e-156	Transducin/WD40 repeat
Sensitive to freezing 6 protein	comp42472_c0_seq4		x		*2.56*	*3.16*		AT4G04920	0.0	SFR6
Transducin wd-40 repeat-containing	comp26403_c0_seq1			x	**–5.97**		**–6.07**	AT3G06880	1e-40	Transducin/WD40 repeat
Kelch repeat-containing f-box family	comp34217_c0_seq1			x	**–5.48**		**–3.97**	AT1G23390	2e-79	Kelch repeat-F-box
Ddb1- and cul4-associated factor	comp43378_c5_seq3			x	**–4.05**		**–2.91**	AT4G31160	0.0	DCAF1 | DDB1-CUL4
Metal-nicotianamine transporter ysl1	comp37162_c0_seq1			x	*2.48*		*1.47*	AT4G24120	0.0	YSL1. ATYSL1
Nudix hydrolase 2-like	comp43278_c0_seq10			x	*2.85*		*3.57*	AT4G12720	4e-89	AtNUDT7. GFG1.

#### Regulation of developmental genes

Numerous down-regulated contigs encoding kinases, A- and D-type cyclins (CYCs), cyclin-dependent kinase (CDK), microtubules, α- and β-tubulins, kinesin, and expansin were found in the three pools considered above, indicating high similarities in the transcript abundance profile of genes involved in SGC and RGC (Supplementary Table S8 at *JXB* online). In the ‘Common pool’, contigs encoding leucine-rich repeat receptor-like protein kinases (comp42644_c0_seq2, comp39844_c0_seq2, comp39539_c0_seq1, and the HAESA-like1 protein homologue comp42887_c1_seq1) were exclusively down-regulated (Table 3). Profiles of transcript abundance of contigs encoding genes involved in cell development were comparable between the three pools, with a total absence of differential expression of these contigs in roots of Pi plants over the whole GC (Fig. 4). Taken together, the transcript abundance profiles over the successive developmental stages illustrate well the common pattern of down-regulation during SGC and RGC. There is also a clear smoothing effect of *P. croceum* on the changes in expression in LRs during _Bto_C transition (Fig. 4). Growth cessation in both leaves and roots is clearly strongly correlated to a down-regulation of transcripts related to C metabolism and cell development.

#### Regulation of hormone-related genes

The up-regulated contig comp43663_c0_seq1 from the ‘Leaf specific pool’ encodes a putative cytokinin transporter with homology to ATPUP1 (Table 3). DECs encoding auxin-responsive factors (ARFs), and auxin efflux and influx carriers, were specific to the ‘Leaf specific pool’ or to the ‘Cont specific pool’, but no DECs were found in the ‘Common pool’, underlining the variability and complexity of the auxin signalling (Supplementary Table S8 at *JXB* online). A homologue (comp7575_c0_seq1) to gibberellin (GA) 20-oxidase was also differentially expressed in the ‘Leaf specific pool’ (Table 3). However, in contrast to auxin- and cytokinin-related genes, GA-related genes were also differentially expressed in the ‘Common pool’. The analyses of the three pools enable differentiation between signalling common to SGC and RGC and signalling specific to leaves in growth cessation.

#### Regulation of transcription factors and other core genes

Log2 FCs of all referred DECs are presented in Supplementary Table S8 at *JXB* online. In the ‘Common pool’, down-regulated transcription factors (TFs) including putative members of RF2, GRAS, and MYB families, and up-regulated members of the PLATZ family were found. More TF contigs were differentially expressed in ‘Cont specific pool’ and ‘Leaf specific pool’. For instance, homologues of the basic helix–loop–helix (bHLH) family such as the bHLH62-like TF (comp34576_c6_seq1) or the bHLH63-like TF (comp29996_c0_seq2) were down-regulated, and homologues of the MYB families were up-regulated. More specifically to the ‘Cont specific pool’, two contigs (comp40603_c0_seq2 and comp22770_c0_seq1) encoding homologues of WRKY TFs were up-regulated, and contigs (comp36477_c0_seq1 and comp22840_c0_seq1) encoding putative GATA TFs and a BZIP TF (comp38285_c0_seq1) were down-regulated (Fig. 4; Table 3).

Further putative candidate core genes were up-regulated in the ‘Common pool’ and encoded a PROLIFERA (PRL) protein (comp39887_c0_seq1), an E3 ubiquitin-protein ligase XBAT31-like1 (comp39201_c1_seq3), and a protein of the MATE efflux family (comp42525_c0_seq1) (Fig. 4; Table 3). Contigs encoding homologues of sensitive to freezing 6 protein (SRF6) (comp42472_c0_seq4) and of metal–nicotianamine transporter YSL1 (comp37162_c0_seq1) were up-regulated in the ‘Cont specific pool’ and in the ‘Leaf specific pool’, respectively (Fig. 4; Table 3). Notably, down-regulated contigs (comp43499_c0_seq2 and comp26403_c0_seq1) encoding predicted ‘transducing WD40 repeat-containing proteins’ were found in the ‘Cont specific pool’ and ‘Leaf specific pool’, and a contig (comp43378_c5_seq3) encoding a ‘DDB1- and CUL4-associated factor homolog 1-like’ protein in the ‘Leaf specific pool’ (Fig. 4). From the 19 DECs found during RGC in control and Pi plants (Cont&Pi_LR intersection), one DEC encoding a predicted cytochrome P450 was also represented in the ‘Cont specific pool’ (Fig. 3). Contigs encoding F-Box KELCH repeat proteins were down-regulated in both the ‘Leaf specific pool’ and ‘Cont specific pool’, and two other KELCH-repeat-containing proteins were up-regulated in the ‘Leaf specific pool’.

## Discussion

Poplar has become a model organism for experimental tree research (Parsons *et al.*, 1986; Tuskan *et al.*, 2006), but it only expresses episodic growth cessation in response to exogenous factors and, as such, does not fit as a model system for endogenous rhythmic growth. Although endogenous control of rhythmic growth was described in oaks more than a century ago (Klebs, 1913), the mechanisms involved have not yet been elucidated. Due to the recalcitrance of trees with endogenous control of growth for *in vitro* technology (McCown, 2000), a tree model system that displays typical endogenous rhythmic growth is still lacking. In this context, genetically identical microcuttings of the oak clone DF159 are highly suitable materials for studying these mechanisms as they display the same alternating shoot and root growth flushes as older saplings (Herrmann *et al.*, 1998).

### ^13^C and ^15^N excess strongly follow the rhythmic growth but only C relative allocation oscillates during rhythmic growth; C and N metabolism are largely organ specific

The findings of high relative C allocation to sink leaves during shoot flush and to growing roots during root flush are consistent with changes observed during episodic growth of *Ligustrum japonicum* cuttings (Kuehny *et al.*, 1997). This finding partially confirms the first hypothesis, as only relative C allocation strongly oscillates during shoot and root flush phases of the rhythmic growth, while shifts in relative N allocation were not significant. Nevertheless, similar drops in the log2 ratios for ^13^C and ^15^N excess as well as for non-structural carbohydrates were observed in the transition from root to shoot flush between stages B and C. Concomitantly with reductions in ^13^C and ^15^N excess in the transition from B to C, strong shifts in DECs were observed in control LRs toward the end of the root elongation phase. Enrichment of GO terms dealing with C and N metabolism in non-inoculated microcuttings differed between leaves and roots and corresponds rather to the specific functions of the two organs, namely photosynthesis in leaves and nutrient uptake in roots. Results of the GO term analyses support the third hypothesis that resource availability may not govern the endogenous rhythmic growth.

### Shoot elongation stage C: the most resource-demanding phase of rhythmic growth cycles

The transient shoot elongation stage C with the lowest biomass appeared to be particularly critical and underlines a dependency of plant biomass on the developmental stage. Accordingly, Palacio *et al.* (2008) observed that the dry matter content of new developed leaves of *Q. faginea* was lowest during the highest shoot elongation. The strong drops in ^13^C and ^15^N excess ratios in LRs as well as of starch in both leaves and LRs during the transition from stage B to C is reflected not only in the lowest LR biomass at stage C, but also in the highest number of down-regulated transcripts in LRs of control plants. The massive transcript down-regulation in LRs during RGC indicates that plants’ physiological efforts are intensely biased towards the above-ground compartment during shoot flushing. At stage C, high resource consumption to build up the new shoot flush is confirmed by enrichment among up-regulated contigs of the leaf-specific GO terms ‘starch metabolic process’ and ‘sucrose transport’, and supports the finding of Le Hir *et al.* (2005*a*) that mobilization of starch by conversion to sucrose in storage tissues supports the strong morphogenic processes during shoot development.

### Impact of *P. croceum*: reduced differential expression to optimize plant energy balance

A further result was the clear demonstration that *P. croceum* suppresses the massive transcript down-regulation during RGC. A further obvious consequence of the *P. croceum* treatment was the suppression of the dramatic reduction in ^13^C and ^15^N excess ratios in leaves and roots in the transition _Bto_C, leading to enhanced plant biomass not only at stage C but across the whole GC. Despite its growth-enhancing effect confirming the previous observation by Herrmann *et al.*, (1998), the fungus did not change the rhythmic growth period of the microcuttings (~27 d in the third GC), which was not very different from the 21 d period described for oak seedlings (Lavarenne, 1966; Champagnat *et al.*, 1986). This supports the second hypothesis: that *P. croceum* affects neither rhythmic growth nor resource allocation patterns between shoots and roots. It indicates that resources are unlikely to be the factors determining alternating rhythmic growth in shoots and roots as previously suggested (Parmentier, 1993; Le Hir *et al.*, 2005*a, b*; Costes *et al.*, 2006).

### Identification of core genes important in endogenous rhythmic growth

The verification of the first two hypotheses supported expectations that core genes regulate the endogenous rhythmic growth common to control and *P. croceum*-treated plants. In accordance with the third hypothesis, analyses of transcriptional changes during SGC and RGC revealed co-ordinated regulation of contig expressed networks involved in microtubule formation, cytoskeleton organization, C metabolism, and hormonal signalling. Further changes involved flowering contigs and others regulated by the circadian clock.

#### Differential expression in C and N metabolism and developmental genes related to alternating growth cessation

The down-regulation of acid invertase and sucrose synthase transcripts specifically in leaves at growth cessation and the high non-structural carbohydrate levels with increased log2 ratios suggest that during bud rest stage A, the leaves are well supported with soluble sugar. This high carbohydrate status is balanced by N acquisition, as indicated by increased ^15^N excess and up-regulated ammonium and nitrate/peptide transporter expression. These results confirm findings of Le Hir *et al.* (2005*a*), that sucrose synthase activity relates to shoot rhythmic growth in *Q. robur* seedlings. On *Q. pubescens*, Willaume and Pages (2011) hypothesized that the response of root growth to areal periodic growth is largely controlled by carbohydrate availability. Signalling mechanisms of sugar have been described (Gibson, 2005; Rolland *et al.*, 2006). However, in the context of endogenous rhythmic growth, a conclusion cannot be reached on a signalling role for carbohydrates. The absence of differential expression of N metabolic core genes in the ‘Common pool’ and the low variations in relative N allocation indicate that N acquisition has a low impact in rhythmic growth. In nature, short days induce growth cessation and bud dormancy (Kayal *et al.*, 2011; Lesur *et al.*, 2015). Similarly to the down-regulation of acid invertase transcripts specific to the ‘Leaf specific pool’, down-regulation of cell wall invertases has been detected during white spruce bud formation under short-day treatment by Kayal *et al.* (2011). These authors also found genes encoding endoglucanases to be down-regulated during bud formation, whereas in the pedunculated oak microcuttings used here, genes associated with cell wall expansion (endoglucanase, xyloglucan endotransglucosylase hydrolase, and expansin) were down-regulated in the ‘Common pool’, demonstrating that cell wall modifications are not exclusive to bud formation under inductive short-day conditions, but are also expressed under long-day conditions during endogenous growth cessation in leaves and roots. Further genes associated with the cell cycle were down-regulated during SGC and RGC. Similar changes including down-regulation of A-, B-, and D-type CYCs and CDK B during meristem inactivation are reportedly involved in poplar bud formation (Ruttink *et al.*, 2007). Furthermore, up-regulation of CYC and CDK genes during oak bud swelling has been observed by Lesur *et al.* (2015). Similarly to the present observation of down-regulated tubulins, reduction in levels of β-tubulin expression during dormancy induction and fast accumulation during dormancy release have been detected in several woody trees including *Q. robur* by Bergervoet *et al.* (1999). Finally, similarities between genes regulated during bud dormancy and differentially expressed genes during endogenous rhythmic growth were not specific to the shoot, as they were also differentially expressed in roots. It is therefore believed that such transcripts involved in cell wall formation and growth cessation play key roles in cell cycling, morphogenesis, and development of plant cells (Vassileva *et al.*, 2005), but are not sufficiently specific to be candidate core genes for signalling roles in endogenous rhythmic growth.

#### Hormone signalling network

GAs have been associated with dormancy release in deciduous trees (Zhuang *et al.*, 2013; Zawaski and Busov, 2014; Lesur *et al.*, 2015). In contrast, reductions in active GA levels are essential for growth cessation, and short-day conditions induce down-regulation of GA20-oxidases (Cooke *et al.*, 2012). In the oak systems employed here, cultivated under long days, the GA20-oxidase was also differentially expressed at SGC. The putative importance of cytokinins during rhythmic growth underlined by Champagnat *et al.* (1986), who demonstrated rhythmic cytokinin production during endogenous rhythmic shoot growth of oak seedlings, was supported by the up-regulation of a candidate cytokinin transporter contig. The down-regulation of auxin-related contigs during growth cessation is consistent with the induced auxin-associated gene expression levels observed during the bud swelling phase of oaks (Lesur *et al.*, 2015). Overall, it can be concluded that the core genes include genes involved in GA and auxin/cytokinin signalling, which could play key roles in endogenous rhythmic growth.

#### Homologues of genes involved in flowering and/or susceptible to be regulated by the circadian clock

The observed enrichment of GO terms associated with flowering, circadian rhythm, and light signalling fits well with increasing evidence that the circadian clock may play important roles in trees during the transition from active bud to dormancy, and flowering (Kozarewa *et al.*, 2010; Olsen, 2010; Cooke *et al.*, 2012; Zhuang *et al.*, 2013; Tylewicz *et al.*, 2015). The present oak transcriptomic data were therefore analysed in relation to the circadian clock framework.

Homologues to TFs such as MYB-like TF, ZING finger TF, bHLH TF, and GATA and bZIP TFs, which are differentially expressed during SGC and RGC in control plants, are also essential for flowering (Huang *et al.*, 2013). Promotion of floral initiation and activation of FT (Flowering locus T) mRNA expression by the CRY2-dependent activity of multiple bHLH proteins is reported by Liu *et al.* (2013). HAESA-like1 protein controls floral organ abscission in *Arabidopsis thaliana* (Takahashi *et al.*, 1998), and *SFR6* is a floral gene that is also transcribed abundantly in *A. thaliana* and *Cary cathayensis* (hickory) during flowering (Huang *et al.*, 2013). In addition, Knight *et al.* (2008) demonstrated that SFR6 is regulated by the circadian clock, as are KELCH-repeat and F-Box 1 (FKF1) proteins which are blue-light photoreceptors that participate in the photoperiodic adjustment of the circadian clock and photoperiodic flowering (Eriksson and Millar, 2003; Chen *et al.*, 2010; Ito *et al.*, 2012). Chen *et al.* (2010) reported that the protein complexes DDB1-CUL4 ASSOCIATED FACTOR1 (DCAF1) and DDB1 binding WD40 (DWD) have functions in photoperiod signalling pathway of *A. thaliana*. Up-regulation of the metal–nicotianamine transporter YSL1-, and e3 ubiquitin-protein ligase XBAT31-encoding contigs indicates a specific role for the leaves in rhythmic growth adjustment.

Finally, in both expression profiles specific to leaves during growth cessation or common to shoot and root growth cessation, homologues to genes known to be involved in flowering but also regulated by the circadian clock in *A. thaliana* were co-regulated and expressed in parallel to the rhythmic growth. These findings would support the hypothesis that the endogenous rhythmic growth in trees is a circadian clock-regulated phenomenon.

In order to better understand how trees develop and adapt to their environment, further improvements of tree models are needed to define which genes control the endogenous rhythmic growth and which may interplay with episodic growth cessation controlled by external factors such as temperature, photoperiod, and drought. The presented oak microcutting system is a new model for this kind of exploration.

## Supplementary data

Supplementary data are available at *JXB* online.

Figure S1. Quantitative real-time PCR of eight contigs.

Figure S2. Frequency of developmental stages during rhythmic growth.

Table S1. Quantitative real-time PCR primers.

Table S2. Statistics of pre-processed paired-end reads; DEC fold changes in the pairwise comparisons

Table S3. DEC repartitions in the intersections and pools.

Table S4. GO terms enriched in the pairwise comparisons.

Table S5. GO terms enriched in the intersections and pools.

Table S6. Number of differentially expressed contigs.

Table S7. Selected enriched GO terms.

Table S8. Log2 FC of selected DECs.

Supplementary Data
