# Supplementary material for: Endogenous rhythmic growth in oak trees is regulated by internal clocks rather than resource availability
Source: J Exp Bot. 2015 Aug 28;66(22):7113–27. doi: 10.1093/jxb/erv408 (PMC4765786; doi:10.1093/jxb/erv408)
Supplement: Supplementary Data [file supp_66_22_7113__index.html]

Endogenous rhythmic growth in oak trees is regulated by internal clocks rather than resource availability — Endogenous rhythmic growth in oak trees is regulated by internal clocks rather than resource availability — Supplementary Data 

# Endogenous rhythmic growth in oak trees is regulated by internal clocks rather than resource availability

## Supplementary Data

Data files

- Supplementary Data - Supplementary Data
- Supplementary Data - Supplementary Data
- Supplementary Data - Supplementary Data
- Supplementary Data - Supplementary Data
- Supplementary Data - Supplementary Data
