# Supplementary material for: Endogenous rhythmic growth in oak trees is regulated by internal clocks rather than resource availability
Source: J Exp Bot. 2015 Aug 28;66(22):7113–27. doi: 10.1093/jxb/erv408 (PMC4765786; doi:10.1093/jxb/erv408)
Supplement: Supplementary Data [file supp_erv408_jexbot137968_file001.pdf]

"Endogenous rhythmic growth in oak trees is regulated by internal clocks rather than resource availability"

Supplementary Data

Supplementary Figure S1: Quantitative real-time PCR (qRT-PCR) confirmation of the differential expression of eight contigs in sink leaves of stage D and source-1 leaves of stage A ($_{Dto}$A) and five in lateral roots (LR) of stages B and C ($_{Bto}$C) of rhythmically growing Oak microcuttings (*Quercus robur* L.). The presented results of RNA sequencing (RNA-Seq, black bars) are means of three biological replicates. qRT-PCR results (orange bars) are means of three biological and two technical replicates, normalized with respect to an 18S rRNA gene. The coefficient of variation was < 6.0 for all qRT-PCR reactions. The analyzed transcripts were predicted to encode the following proteins by BLASTx searches against the NCBI nr database at an E-value cut-off of 6.0e–41: A, farnesene synthase (A), chalcone synthase (B), type-a response regulator (C), GDSL esterase/lipase (D), acyl-CoA synthetase 1 (E), ring-u box domain containing protein (F), unknown protein (G), asparagine synthetase (H), catalase (I), leucine-rich receptor-like protein kinase (J), and lipid transfer protein (K).

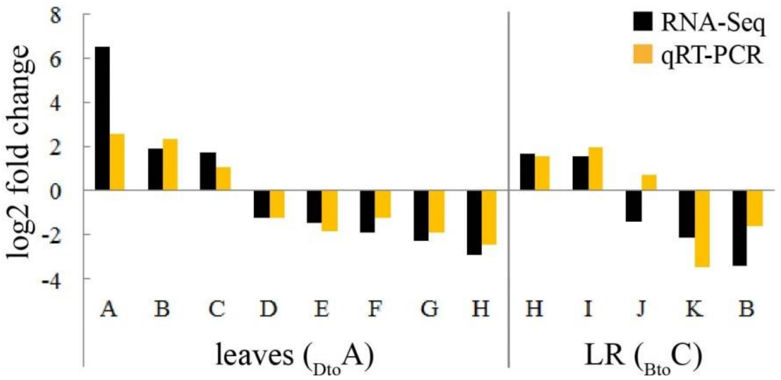

Supplementary Figure S2: Frequency of the successive developmental stages of the terminal shoot flush in **(A)** control and **(B)** *Piloderma croceum*-inoculated *Q. robur* microcuttings. ■ stage A, ■ stage B, ■ stage C, ■ stage D, Plant numbers are given in brackets. Shoot and root dry weight (DW) at the successive developmental stages of **(C)** control and **(D)** *Piloderma croceum*-inoculated plants after eight weeks (mean ± SE). The number-letter combinations refer to the growth cycle (GC) number and developmental stage, e.g. 1A refers to GC1 in developmental stage A. One growth cycle comprises alternating shoot flushing (SF) and root flushing (RF). **(E)** Sets of plants used for analyses according to their developmental stages A, B, C and D.

Figure S2

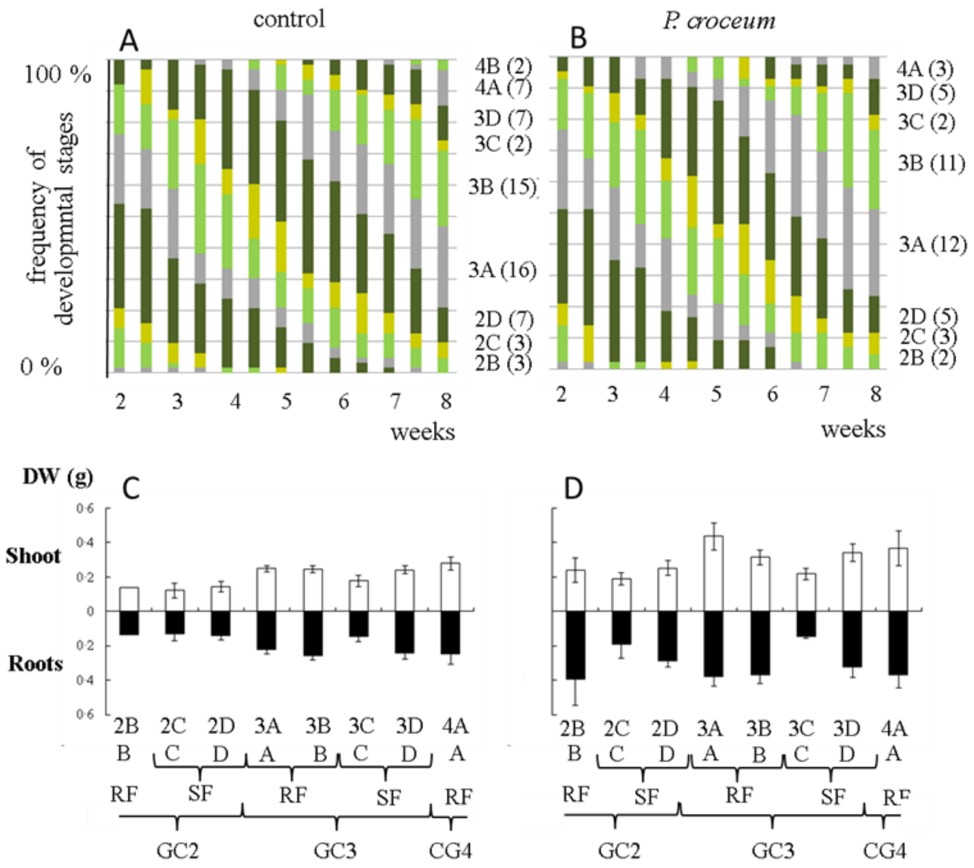

E

	Root flush		Shoot flush		
	Stage A	Stage B	Stage C	Stage D	Total
Control	22	18	11	14	65
P. croceum	15	10	8	9	42
Total	37	28	19	23	

Supplementary Table S1: Fold change of DECs in the pairwise comparisons **Excel file S1**

Supplementary Table S2: Quantitative real-time PCR primers. We used BLASTx searches against the NCBI nr database to predict gene function.

Contig_no.	Primer_ID	Primer_sequence	Predicted gene function	Alignment e-value; organism giving the best BLASTx match
43737_c0_seq2	WP0-43737_f	GAAAGCAAGGCCGACTGAGC	α-Farnesene synthase	4e-136; *Ricinus communis*
	WP0-43737_r	GGTGGTGGAGAAATCTGGGG		
33862_c0_seq1	WP0-33862_f	GCTCGCTCTTGGTGATTCGG	Chalcone synthase	6e-41; *Populus alba*
	WP0-33862_r	AGCCACTCCCTCCAACTGTG		
28471_c0_seq2	WP0-28471_f	GCCTGGAATGACTGGCTACG	Type-a response regulator	1e-66; *Vitis vinifera*
	WP0-28471_r	CTCTGCCCCTTCCTCCAAAC		
30487_c1_seq1	WP0-30487_f	GGCCGAATTGCCACAGAC	GDSL esterase/lipase	1e-88; *Vitis vinifera*
	WP0-30487_r	GTCATAGCCAGTCCCAGCAG		
39617_c0_seq1	WP0-39617_f	GAGCCAGGGGCAGGAAAGAA	Long chain acyl-CoA synthetase 1	0; *Glycine max*
	WP0-39617_r	GTGGCACGAGTGGAGATCCT		
34555_c1_seq1	WP0-34555_f	GTCTGCTGCTGCTCTATGGG	Ring u-box domain-containing protein	1e-144; *Vitis vinifera*
	WP0-34555_r	GATTCTTCGCTGCCCCTCTG		
43489_c1_seq2	WP0-43489_f	CGGTTGTTAGGTTGGGGAGC	Unknown protein	0; *Populus trichocarpa*
	WP0-43489_r	CCCACTCCACTAATCCTGCG		
36880_c0_seq1	WP0-36880_f	CTTCCAGTCCCCAAGCAGAG	Asparagine synthetase	0; *Populus trichocarpa*
	WP0-36880_r	CCACAAGGCACCCAACAAGG		
32087_c0_seq1	WP0-32087_f	TTCTTGCCTGTCTGGTGCCC	Catalase	0; *Ziziphus jujuba*
	WP0-32087_r	CGTCCTGCTGAAAGGTTCCC		
41108_c1_seq1	WP0-41108_f	GCTACCCCAAAGTCGGCA	Leucine-rich receptor-like protein kinase	0; *Populus trichocarpa*
	WP0-41108_r	GGGAGCGGCAAAGGGAAT		
30052_c0_seq1	WP0-30052_f	GCCAAGTCCAAAGCAGCC	Lipid transfer protein	4e-60; *Castanea sativa*
	WP0-30052_r	GCAAGCCTGTGTGGGAGA		

Supplementary Table S3: DECs repartition in the intersections and pools **Excel file S3**

Supplementary Table S4: GO terms enriched in the pairwise comparisons **Excel file S4**

Supplementary Table S5: GO terms enriched in the intersections **Excel file S5**

Supplementary Table S6: Numbers of differentially expressed contigs (total, up-regulated, down-regulated) in the developmental stage pairwise comparisons D to A ($_{Dto}$A), A to B ($_{Ato}$B) *etc*. of leaves (Cont_Leaf, Pi_Leaf) and roots (Cont_LR, Pi_LR) of control (Cont) and *P. croceum* inoculated (Pi) *Q. robur* L microcuttings, and tissue specific intersections of control and inoculation related comparisons (Cont&Pi_Leaf, Cont&Pi_LR).

	**Number of DE contigs in leaves**			**Number of DE contigs in lateral roots**		
	**Total**	**Up**	**Down**	**Total**	**Up**	**Down**
		**Cont_Leaf**			**Cont_LR**	
$_{Dto}$**A**	3138	1196	1956	35	13	22
$_{Ato}$**B**	73	3	70	5	1	4
$_{Bto}$**C**	11	8	3	4353	1751	2602
$_{Cto}$**D**	2	1	1	529	415	114
		**Pi_Leaf**			**Pi_LR**	
$_{Dto}$**A**	1144	284	860	37	7	30
$_{Ato}$**B**	741	495	246	15	7	8
$_{Bto}$**C**	365	323	42	37	30	7
$_{Cto}$**D**	14	6	8	7	4	3
		**Cont&Pi_Leaf**			**Cont&Pi_LR**	
$_{Dto}$**A**	770	178	592	3	0	3
$_{Ato}$**B**	29	1	28	0	0	0
$_{Bto}$**C**	0	0	0	19	18	1
$_{Cto}$**D**	0	0	0	2	1	1

Supplementary Table S7: Log10 *p*-values for selected enriched Gene Ontology (GO) terms associated with carbon and nitrogen metabolism, cell development, hormone signaling and other functions. Enriched GO terms among up- and down-regulated contigs detected in the three pairwise comparisons D to A ($_{Dto}$A) for leaves during shoot growth cessation in control (Cont_Leaf) and in *P. croceum* treated *Q. robur* L. microcuttings (Pi_Leaf) and pairwise comparisons B to C ($_{Bto}$C) in control lateral roots (Cont_LR) during root growth cessation, (Green color for GO terms enriched in down-regulated genes; orange color for GO terms enriched in up-regulated genes). Enriched GO terms found for the intersections Cont&Pi_Leaf and Cont_Leaf&LR and for their “Common pool” Cont&Pi_Leaf∩Cont_Leaf&LR of DECs (Blue for GO terms enriched in down-regulated genes and red for GO terms enriched in up-regulated genes).

Go_ID		Cont Leaf $_{Dto}$A	Cont LR $_{Bto}$C	Pi Leaf $_{Dto}$A	"Common pool"	Inter-section Cont Leaf&LR	Inter-section Cont&Pi Leaf
**C Metabolism**							
GO:0009225	nucleotide-sugar metabolic process	-5,40	-2,29	-7,19	-5,93		-2,40
GO:0003979	UDP-glucose 6-dehydrogenase activity	-3,16	-2,67	-4,10	-5,69		
GO:0000271	polysaccharide biosynthetic process	-6,69	-5,39	-3,40	-4,67	-2,79	
GO:0048040	UDP-glucuronate decarboxylase activity	-3,40	-6,50	-4,61	-4,64		
GO:0005975	carbohydrate metabolic process	-3,35	-2,70	-5,44	-4,00		-1,66
GO:0009749	response to glucose			-2,34	-2,24		
GO:0009744	response to sucrose	2,25	6,92	-1,53	-1,76	1,89	
GO:0015786	UDP-glucose transport				-1,42		
GO:0000271	polysaccharide biosynthetic process					-2,79	
GO:0005985	sucrose metabolic process	-2,37		-4,04			-4,52
GO:0009969	xyloglucan biosynthetic process						-2,66
GO:0004575	sucrose alpha-glucosidase activity						-1,88
GO:0010131	sucrose catabolic process, using invertase or sucrose synthase						-1,37
GO:0015770	sucrose transport	2,50		1,85			1,85
GO:0005982	starch metabolic process			1,89			1,97
GO:0010037	response to carbon dioxide	1,62					
GO:0010109	regulation of photosynthesis	2,41					
GO:0009643	photosynthetic acclimation	2,56					
GO:0015760	glucose-6-phosphate transport	3,31					
GO:0019253	reductive pentose-phosphate cycle		-1,61				
GO:0043617	cellular response to sucrose starvation		1,41				
GO:0009311	oligosaccharide metabolic process		2,03				
GO:0015976	carbon utilization		3,70				
GO:0005992	trehalose biosynthetic process		5,51				
**N Metabolism**							
GO:0010167	response to nitrate			-1,48	-1,33		
GO:0006995	cellular response to nitrogen starvation	4,20		2,69		1,32	2,05
GO:0009308	amine metabolic process	-2,05		-4,69			-3,49
GO:0019344	cysteine biosynthetic process		-3,46				
GO:0019853	L-ascorbic acid biosynthetic process		-3,32				
GO:0006571	tyrosine biosynthetic process		-2,66				
GO:0071731	response to nitric oxide		-2,02				
GO:0042398	cellular modified amino acid biosynthetic process		-1,96				
GO:0070981	L-asparagine biosynthetic process		1,41				
GO:0006809	nitric oxide biosynthetic process		1,52				
GO:0006541	glutamine metabolic process		2,18				
GO:0019477	L-lysine catabolic process		2,76				
GO:0006552	leucine catabolic process		3,13				
**Development**							
GO:0007018	microtubule-based movement	-28,15	-29,58	-13,69	-20,43	-16,74	
GO:0010075	regulation of meristem growth	-14,35	-13,19	-4,80	-8,96	-5,18	
GO:0009664	plant-type cell wall organization	-10,06	-12,11	-8,08	-6,95	-1,95	-2,44
GO:0008361	regulation of cell size	-4,26	-2,57	-4,43	-5,65		
GO:0007020	microtubule nucleation	-3,59	-6,09	-3,02	-4,26		
GO:0009825	multidimensional cell growth	-7,19	-5,43	-1,39	-4,23	-1,74	
GO:0008283	cell proliferation	-8,93	-16,48	-2,03	-4,08	-9,11	
GO:0048653	anther development	-3,01	-3,36	-1,86	-3,57		
GO:0009932	cell tip growth	-6,78	-5,75	-2,19	-3,54	-3,65	
GO:0000226	microtubule cytoskeleton organization	-6,81	-4,18	-1,39	-2,72	-5,00	
GO:0000904	cell morphogenesis involved in differentiation	-1,38	no	-1,68	-2,18		
GO:0007010	cytoskeleton organization	-1,60	-5,24	-1,40	-2,01		
GO:0042545	cell wall modification	-7,09	-2,30	-3,94	-1,90	-1,51	-2,59
GO:0071258	cellular response to gravity	-2,43	-3,62	-1,34	-1,87	-1,73	
GO:0051301	cell division	-2,58	-6,59		-1,74	-2,08	
GO:0000911	cytokinesis by cell plate formation	-7,48	-8,13	-2,33	-1,68	-6,64	
GO:0051726	regulation of cell cycle	-6,94	-7,88			-8,44	
GO:0006270	DNA replication initiation	-5,68	-9,04	-1,74		-6,34	
GO:0051445	regulation of meiotic cell cycle	-1,77		-2,38			-3,20
GO:0007047	cell wall organization	-3,65	-3,22	-2,55			-3,01
GO:0030041	actin filament polymerization	-2,25					-1,65
GO:0000280	nuclear division	-1,79	-2,90	-1,93			-1,34

Journal of Experimental Botany – S. Herrmann, S. Recht, M. Boenn, L. Feldhahn. O. Angay, F. Fleischmann, M. T. Tarkka, T.E.E. Grams, F. Buscot. 2015,
Endogenous rhythmic growth in oaks trees, Supplementary Data

**Hormone signaling**							
GO:0009740	gibberellic acid mediated signaling pathway	-1,82		-2,30	-2,89		
GO:0006564	L-serine biosynthetic process			-1,41	-2,37		
GO:0006569	tryptophan catabolic process			1,61	-2,13		1,69
GO:0009926	auxin polar transport			-2,02	-2,11		
GO:0006555	methionine metabolic process		-2,46	-3,43	-1,91		
GO:0009695	jasmonic acid biosynthetic process				1,42		
GO:0010817	regulation of hormone levels	-4,53	-3,82			-2,51	
GO:0009751	response to salicylic acid	1,43	2,94			1,85	
GO:0009734	auxin mediated signaling pathway	-5,34					-2,35
GO:0010011	auxin binding	-1,60					-1,33
GO:0080151	positive regulation of salicylic acid mediated signaling pathwa	1,65		2,83	.		1,49
GO:0042430	indole-containing compound metabolic process			1,64			1,68
GO:0010184	cytokinin transport	1,70		2,31			2,55
**Others**							
GO:0016682	oxidoreductase activity, acting on diphenols and related substa	-2,39	-5,03	-1,90	-1,90	-2,75	
GO:0048451	petal formation	-4,04	-2,58	-2,06	-1,47	-3,02	-1,41
GO:0005507	copper ion binding	-8,49	-15,77	-5,93	-1,36	-2,96	-1,52
GO:0009835	fruit ripening	1,45			1,97		
GO:0016705	oxidoreductase activity, acting on paired donors,	1,46		1,52	2,79		
GO:0003939	L-iditol 2-dehydrogenase activity	3,07	2,86	2,05	3,16	2,60	
GO:0048449	floral organ formation	-2,76	-1,97			-4,44	
GO:0009909	regulation of flower development					-3,42	
GO:0009908	flower development					-1,59	
GO:0048579	negative regulation of long-day photoperiodism, flowering				.	-1,31	
GO:0009631	cold acclimation					1,38	
GO:0032922	circadian regulation of gene expression		2,09			1,86	
GO:0048586	regulation of long-day photoperiodism, flowering					2,29	
GO:0010161	red light signaling pathway			1,31			1,35
GO:0048316	seed development			1,62			1,70
GO:0048653	anther development			1,82			1,90

Supplementary Table S8: Log2 FC of selected DE contigs (Benjamin-Hochberg-adjusted *p*-values < 0.01) dealing with carbon and nitrogen metabolism, cell development, hormone signaling and other functions in pairwise comparisons between D to A transition ($_{Dto}A$) for leaves in control (Cont_Leaf) and in *P. croceum* treated *Q. robur* microcuttings (Pi_Leaf) at shoot growth cessation and pairwise comparisons between B to C transition ($_{Bto}C$) in control lateral roots (Cont_LR) at root growth cessation. Presence (x) is given for these DECs in the three pools resulting from the intersections Cont&Pi_Leaf and Cont_Leaf&LR at shoot and root growth cessation (*p*-values < 0.01): in the "Common pool" differentially expressed contigs (DECs) are common to LR and leaves of controls and leaves of inoculated plants, in the "Leaf specific pool" DECs are common to control and inoculated leaves, in the "Cont specific pool" DECs are common to leaves and LR of control plants. Contig descriptions originate from Blast2GO annotation of the OakContigDF159.1 reference library (Tarkka et al. 2013). Down-regulated contigs are in blue and up-regulated in red.

		"Common pool"	"Control specific pool"	"Leaf specific pool"	Cont Leaf DtoA	Cont LR BtoC	Pi Leaf DtoA
**C metabolism**							
endo-beta- -glucanase	comp36907_c0_seq1	x			-6,05	-2,11	-4,98
xyloglucan endotransglucosylase hydrolase protein a	comp21531_c0_seq1	x			-4,60	-3,18	-3,69
endoglucanase 17 (GH17)	comp35144_c0_seq3	x			-4,50	-1,86	-3,64
probable polygalacturonase non-catalytic subunit jp650	comp43358_c0_seq1	x			-4,45	-2,15	-3,37
xyloglucan endotransglycosylase	comp19836_c0_seq1	x			-4,26	-3,21	-4,11
glucan endo- -beta-glucosidase (GH71)	comp28162_c0_seq1	x			-4,20	-2,25	-3,42
glycosyl hydrolase family 9 (GH9)	comp37579_c1_seq1	x			-4,10	-2,61	-3,82
beta-galactosidase 3-like	comp42471_c1_seq4	x			-4,03	-2,54	-3,82
cellulose synthase a catalytic subunit 3	comp35922_c0_seq1	x			-3,61	-3,01	-2,38
endo-beta- -glucanase (GH26)	comp35144_c0_seq1	x			-2,73	-1,42	-2,18
glycoside hydrolase family 28 protein (GH28)	comp35609_c0_seq1	x			-2,45	-1,51	-2,02
lysosomal beta glucosidase-like (GH3)	comp29816_c0_seq1	x			-2,34	-3,05	-1,90
pectin methylesterase	comp43510_c0_seq1	x			-2,19	-1,37	-1,95
glucan endo- -beta-glucosidase	comp28162_c0_seq2	x			-2,08	-1,86	-2,01
glucan endo- -beta-glucosidase (GH71)	comp40912_c0_seq1	x			-2,05	-1,96	-1,98
pectin methylesterase	comp29797_c0_seq1	x			-1,93	-1,20	-1,98
xylan 1 4-beta-xylosidase (GH43)	comp42261_c0_seq2	x			-1,80	-2,05	-2,07
udp-glucuronic acid decarboxylase 1	comp34100_c1_seq1	x			-1,79	-1,74	-1,36
o-glycosyl hydrolases family 17 protein (GH17)	comp41738_c0_seq1	x			-1,70	-2,45	-1,76
udp-d-glucuronic acid 4-epimerase	comp38512_c0_seq1	x			-1,41	-1,50	-1,30
udp-glucose dehydrogenase	comp34020_c0_seq1	x			-1,41	-1,92	-1,88
udp-xyl synthase 5	comp37662_c0_seq1	x			-1,29	-1,40	-1,78
udp-glucuronic acid decarboxylase 1	comp34100_c0_seq1	x			-1,27	-1,17	-1,37
endo- -beta-glucanase (GH9)	comp35074_c0_seq1	x			-1,27	-0,95	-1,15
udp-glucose 6-	comp35195_c0_seq1	x			-1,12	-1,16	-1,52
beta- -glucanase	comp44202_c0_seq1		x		-2,46	-1,56	
nodulin-like protein	comp40007_c0_seq1		x		-2,03	-1,32	
glycosyl hydrolase family 3 protein (GH3)	comp42755_c0_seq1		x		-1,18	-0,86	
udp-glycosyltransferase 75d1-like	comp43576_c0_seq4		x		0,91	1,96	
glucan endo- -beta-glucosidase 14-like	comp40819_c0_seq1		x		2,91	1,70	
endo- - -beta-d-	comp40668_c1_seq14		x		4,00	2,93	
acid invertase	comp37785_c0_seq1			x	-6,22		-4,32
sucrose synthase	comp28647_c0_seq1			x	-6,00		-4,39
udp-glycosyltransferase 91a1-like	comp30949_c1_seq1			x	-4,19		-5,23
polygalacturonase-like protein	comp38500_c0_seq1			x	-3,30		-3,27
beta-galactosidase 3	comp38546_c1_seq1			x	-3,00		-2,95
sucrose synthase sus1	comp32110_c0_seq1			x	-2,32		-2,24
o-glycosyl hydrolases family 17 protein (GH17)	comp36698_c0_seq1			x	-2,29		-2,29
glycine decarboxylase complex h-protein	comp40767_c0_seq2			x	-1,61		-2,16
mannan endo- -beta-mannosidase 2-like	comp28518_c0_seq1			x	-1,47		-1,26
glycerol-3-phosphate dehydrogenase	comp38213_c0_seq1			x	-1,46		-1,62
ribonuclease j-like	comp42043_c0_seq1			x	1,10		0,96
monosaccharide-sensing protein	comp41353_c0_seq3			x	1,17		1,07
endo- - -beta-d-	comp40668_c1_seq10			x	1,23		1,00
glutathione s-transferase	comp42369_c1_seq1			x	1,30		0,82
udp-glucose pyrophosphorylase 3	comp28714_c0_seq1			x	1,36		1,32
sucrose-phosphatase 2	comp41528_c0_seq1			x	1,48		0,91
udp-glycosyltransferase 85a1-like	comp32006_c0_seq2			x	1,50		1,51
beta glucosidase 11	comp43151_c1_seq2			x	1,84		1,03
nodulin 3 family protein (Sweet12)	comp40679_c0_seq1			x	2,03		3,03
protein tyrosine expressed	comp38954_c0_seq1			x	2,34		1,59
**N metabolism**							
threonine aldolase	comp37681_c0_seq1		x		0,88	2,61	
ammonium transporter amt2	comp38427_c0_seq1		x		1,77	1,29	
probable peptide nitrate transporter at5g62680-like	comp43246_c1_seq1		x		1,84	2,27	
urea active transporter-like protein	comp43623_c2_seq1			x	0,90		1,10

		"Common pool"	"Control specific pool"	"Leaf specific pool"	Cont Leaf $_{Dto}$A	Cont LR $_{Bto}$C	Pi Leaf $_{Dto}$A
**Development**							
leucine-rich repeat receptor-like protein kinase pxl2-lik	comp42644_c0_seq2	x			-5,05	-3,90	-6,42
receptor-like kinase	comp35210_c0_seq1	x			-4,60	-2,90	-3,18
leucine-rich repeat receptor-like protein kinase	comp39844_c0_seq2	x			-2,73	-1,39	-2,26
receptor protein kinase clavata1	comp38911_c1_seq1	x			-2,47	-0,98	-1,91
leucine-rich repeat receptor-like protein kinase	comp39539_c0_seq1	x			-2,22	-1,53	-2,14
kinase-like protein tmkl1-like	comp39900_c1_seq1	x			-1,60	-1,34	-1,70
leucine-rich repeat receptor-like protein kinase	comp42887_c1_seq1	x			-1,04	-1,22	-1,19
cell division cycle cofactor of apc complex	comp39991_c1_seq3	x			-2,74	-2,57	-2,33
cell division control protein 45 homolog	comp38086_c0_seq1	x			-2,60	-2,65	-2,87
cyclin a2	comp41558_c0_seq1	x			-3,43	-2,40	-3,17
cyclin d3	comp36768_c4_seq1	x			-3,25	-1,21	-2,57
cyclin dependent kinase b	comp40802_c0_seq1	x			-3,02	-2,86	-2,09
microtubule-associated protein spiral2-like	comp37487_c0_seq1	x			-3,69	-2,50	-2,44
α-tubulin 1	comp40811_c2_seq1	x			-3,67	-2,92	-2,69
atp binding microtubule motor	comp37551_c0_seq1	x			-3,19	-3,07	-2,70
α-tubulin 6 chain	comp30627_c1_seq1	x			-2,67	-1,71	-2,70
β-tubulin-2 -3	comp36418_c0_seq1	x			-2,49	-2,54	-2,06
ß-tubulin beta	comp41921_c1_seq11	x			-2,15	-2,03	-2,19
β-tubulin	comp30053_c0_seq1	x			-1,25	-2,12	-1,39
kinesin-like protein	comp33147_c0_seq1	x			-3,70	-2,32	-2,12
kinesin-like protein kif3a	comp40573_c0_seq1	x			-3,39	-2,67	-2,44
phragmoplast-associated kinesin-related protein	comp42517_c0_seq1	x			-3,30	-3,24	-4,00
bipolar kinesin krp-	comp34041_c0_seq1	x			-3,17	-2,81	-1,98
kinesin-like protein kif2a-like	comp40417_c0_seq1	x			-3,06	-2,02	-2,56
kinesin-like protein	comp42559_c1_seq6	x			-2,68	-0,95	-2,26
kinesin family member 2 24	comp24923_c0_seq1	x			-1,04	-0,88	-0,98
expansin-b3-like precursor	comp38597_c0_seq1	x			-6,08	-2,61	-5,57
expansin	comp43870_c0_seq1	x			-4,43	-1,15	-4,81
expansin	comp40685_c0_seq1	x			-3,54	-1,38	-2,85
expansin	comp43378_c2_seq1	x			-2,22	-1,66	-2,41
thaumatin-like protein	comp43430_c0_seq3	x			-4,91	-3,21	-4,05
kinase like protein	comp42614_c0_seq3		x		-6,38	-8,11	
receptor-like protein kinase	comp42644_c0_seq1		x		-5,94	-3,38	
leucine-rich repeat receptor-like protein kinase pxl2-lik	comp42644_c1_seq1		x		-4,00	-3,72	
kinase like protein	comp42614_c0_seq2		x		-3,06	-3,15	
probable receptor protein kinase tmk1-like	comp28822_c1_seq1		x		-2,67	-1,43	
protein kinase pvpk-1-like	comp37938_c1_seq1		x		-2,53	-1,64	
receptor protein kinase clavata1	comp39300_c1_seq1		x		-2,03	-1,04	
receptor protein kinase-like protein	comp33560_c0_seq1		x		-1,83	-2,02	
receptor-like protein kinase	comp30632_c0_seq1		x		-1,06	-1,23	
cyclin-dependent kinase	comp35140_c0_seq3		x		-4,92	-4,48	
tubulin alpha-6 chain	comp40811_c0_seq1		x		-4,19	-2,81	
microtubule-associated protein rp eb family member 1-	comp35261_c0_seq1		x		-3,49	-2,58	
mitogen-activated protein kinase	comp38266_c0_seq1		x		-2,88	-2,82	
atp binding microtubule motor family protein	comp32645_c0_seq1		x		-2,16	-1,38	
alpha tubulin 1	comp38166_c0_seq2		x		-2,03	-1,51	
microtubule-associated protein tortifolia1	comp33352_c0_seq1		x		-1,11	-1,03	
microtubule associated protein type 2	comp22730_c0_seq1		x		-1,10	-0,92	
kinesin motor family protein	comp43697_c1_seq1		x		-7,90	-7,35	
chromosome-associated kinesin	comp42577_c0_seq1		x		-4,32	-3,46	
kinesin-like protein kif18b-like	comp42577_c0_seq5		x		-4,30	-3,69	
kinesin-like protein kif22-like	comp41675_c0_seq2		x		-3,81	-3,07	
kinesin heavy	comp27811_c0_seq1		x		-3,09	-1,79	
kinesin heavy	comp42763_c0_seq1		x		-3,01	-2,40	
kinesin-related protein	comp35850_c0_seq1		x		-2,93	-1,89	
kinesin-like protein	comp40426_c0_seq1		x		-2,81	-2,77	
kinesin heavy	comp44045_c0_seq1		x		-2,61	-1,20	
kinesin light	comp40582_c0_seq1		x		-1,91	-1,90	
kinesin-like protein	comp39767_c0_seq1		x		-1,80	-2,45	
kinesin-like protein	comp35444_c0_seq1		x		-1,75	-1,63	
kinesin like protein	comp34408_c0_seq1		x		-1,58	-2,54	
kinesin light	comp38101_c1_seq1		x		-1,39	-1,67	
myosin heavy embryonic smooth muscle	comp33008_c1_seq1		x		-1,33	-2,30	
alpha-expansin 4	comp32906_c0_seq1		x		-1,86	-2,13	

		"Common pool"	"Control specific pool"	"Leaf specific pool"	Cont Leaf DtoA	Cont LR BtoC	Pi Leaf DtoA
protein kinase	comp31723_c0_seq1			x	-6,36		-8,72
wall-associated receptor kinase 2-like	comp42903_c0_seq1			x	-2,26		-2,57
receptor-like protein kinase	comp41705_c1_seq1			x	-2,00		-1,67
receptor-like protein kinase	comp43255_c0_seq2			x	-1,91		-1,80
cyclin a-like protein	comp41670_c0_seq2			x	-8,93		-7,26
cyclin a-like protein	comp41670_c0_seq1			x	-8,50		-8,40
d6-type cyclin	comp33413_c0_seq1			x	-8,19		-7,72
cyclin-dependent protein	comp32700_c1_seq1			x	-4,98		-3,18
cyclin-dependent kinase inhibitor 1-like	comp36674_c1_seq1			x	-1,60		-1,67
microtubule-associated protein spiral2-like	comp38813_c0_seq1			x	-1,73		-1,71
kinesin heavy	comp27811_c2_seq1			x	-2,90		-2,34
kinesin light chain	comp40582_c1_seq1			x	-2,10		-1,78
kinesin motor protein	comp41315_c1_seq1			x	-1,53		-0,97
alpha-expansin 8	comp34518_c1_seq2			x	-5,32		-6,27
alpha-expansin 5	comp26430_c0_seq1			x	-5,27		-5,47
alpha-expansin 8	comp34518_c0_seq1			x	-4,52		-6,39
pollen ole e 1 allergen and extensin family protein	comp17691_c0_seq1			x	-7,13		-6,43
pollen ole e 1 allergen and extensin family protein	comp41762_c0_seq1			x	-3,99		-4,18
thaumatin-like protein	comp29882_c0_seq1			x	-4,02		-3,56
**Homone signaling**							
gibberellin-regulated protein	comp29599_c0_seq1	x			-6,58	-3,49	-7,59
gibberellin-regulated protein 1	comp34711_c0_seq1	x			-4,17	-3,78	-3,70
gibberellin receptor	comp30631_c0_seq1		x		1,36	0,89	
gibberellin 20-oxidase	comp7575_c0_seq1			x	-6,86		-6,43
gibberellin induced protein	comp35611_c0_seq1			x	-4,92		-4,45
auxin influx carrier component	comp38228_c2_seq1		x		-8,59	-2,13	
auxin influx carrier component	comp34375_c0_seq1		x		-2,55	-1,55	
auxin-regulated protein	comp23315_c0_seq1		x		-2,03	-1,92	
auxin-responsive protein	comp32281_c1_seq1		x		-1,46	-1,73	
auxin-induced protein 5ng4-like *MtN21 nodulin family* t	comp37627_c0_seq2			x	-10,38		-9,53
auxin-induced protein	comp41946_c0_seq3			x	-5,92		-6,65
auxin influx carrier component	comp29823_c1_seq1			x	-3,38		-2,53
auxin efflux carrier	comp37248_c1_seq1			x	-2,26		-1,75
auxin efflux carrier component	comp38167_c0_seq1			x	-2,23		-2,27
transport inhibitor response 1	comp34179_c1_seq1			x	-1,65	2,69	-1,88
auxin response factor 6-like	comp19485_c0_seq1			x	-0,96		-0,86
purine permease	comp43663_c0_seq1			x	1,62		1,37
**Zinc proteins**							
zinc finger ccch domain-containing protein 15	comp40659_c2_seq1	x			-2,86	-2,50	-2,81
c2h2-type zinc finger protein	comp38564_c2_seq1	x			-2,09	-1,44	-1,99
zinc finger ccch domain-containing protein 53-like	comp42976_c1_seq3		x		-2,41	-1,97	
zinc finger protein	comp41423_c0_seq1		x		1,05	1,82	
zinc finger	comp35351_c0_seq1			x	-10,22		-10,15
zinc finger	comp27443_c0_seq1			x	-7,87		-5,08
zinc finger	comp39125_c0_seq1			x	-7,83		-9,17
zinc finger	comp28752_c1_seq1			x	-5,31		-5,73
zinc finger	comp33248_c0_seq1			x	-4,75		-4,30
zinc finger ccch domain-containing protein 15	comp39647_c0_seq1			x	-3,93		-3,89
zinc finger	comp29675_c0_seq1			x	-1,91		-1,70
gata type zinc finger transcription factor-like protein	comp44130_c0_seq1			x	-1,66		-1,58

		"Common pool"	"Control specific pool"	"Leaf specific pool"	Cont Leaf DtoA	Cont LR BtoC	Pi Leaf DtoA
**Oxido-Reduction**							
cytochrome p450	comp43218_c0_seq4	x			-7,24	-3,57	-8,36
l-idonate 5-dehydrogenase GrosES-like	comp38136_c0_seq1	x			0,89	2,70	0,89
cytochrome p450	comp40876_c0_seq1	x			1,41	1,15	1,31
cytochrome p450 82c4	comp36393_c1_seq1	x			2,00	2,26	2,32
cytochrome p450	comp42884_c2_seq5	x			2,84	1,84	1,70
cytochrome p450	comp40876_c0_seq2		x		1,59	1,68	
cytochrome p450 82a3-like	comp42662_c2_seq2		x		2,54	1,56	
2-oxoglutarate-dependent dioxygenase	comp40403_c0_seq7		x		3,54	2,14	
cytochrome p450	comp38658_c0_seq1			x	-8,92		-8,11
cytochrome p450 86a2-like	comp244042_c0_seq1			x	-6,98		-6,56
cytochrome p450 86a2	comp28615_c0_seq1			x	-6,01		-6,44
cytochrome p450	comp39819_c0_seq1			x	-4,09		-5,91
cytochrome family subfamily polypeptide 4	comp19970_c0_seq1			x	-3,52		-3,11
cytochrome c biogenesis protein precursor	comp35984_c0_seq1			x	1,06		0,99
cytochrome p450	comp40013_c0_seq4			x	2,28		2,89
cytochrome p450	comp42792_c1_seq1			x	2,43		2,11
cytochrome p450	comp41034_c0_seq1			x	2,70		1,87
cytochrome p450	comp43218_c0_seq10			x	2,71		3,69
cytochrome p450	comp29503_c0_seq1			x	3,39		3,46
**Transcription factors**							
transcription factor rf2b	comp36095_c0_seq1	x			-6,64	-3,00	-6,02
gras family transcription factor	comp39403_c0_seq1	x			-3,15	-1,75	-2,54
gras family transcription factor	comp35886_c0_seq1	x			-2,15	-1,36	-2,04
myb-related protein 3r-1-like	comp43723_c0_seq1	x			-1,24	-1,01	-1,02
platz transcription factor domain-containing protein	comp43527_c1_seq3	x			1,48	2,73	1,66
transcription factor rf2b	comp36224_c1_seq1		x		-5,78	-3,81	
transcription factor tcp4-like	comp27734_c0_seq1		x		-5,15	-3,65	
myb-related protein 3r-1-like	comp26844_c0_seq1		x		-4,27	-2,65	
transcription factor rf2b	comp34714_c2_seq1		x		-3,65	-1,73	
myb-related protein 3r-1-like	comp27947_c1_seq1		x		-3,55	-3,05	
heat stress transcription factor b-4	comp33275_c0_seq1		x		-3,45	-2,43	
e2f transcription factor-like e2fe-like	comp42759_c0_seq2		x		-3,41	-2,66	
gata transcription factor	comp36477_c0_seq1		x		-3,23	-2,95	
transcription factor bhlh49	comp37639_c0_seq1		x		-2,71	-1,54	
bzip transcription factor bzip133	comp40370_c0_seq1		x		-2,66	-1,14	
transcription factor rf2b	comp34714_c1_seq1		x		-2,43	-1,91	
bzip protein	comp38105_c0_seq1		x		-2,33	-2,25	
heat stress transcription factor b-4	comp35650_c0_seq1		x		-2,19	-2,49	
transcription factor bhlh62-like	comp34576_c0_seq1		x		-2,01	-1,15	
transcription factor bpe-like	comp34562_c0_seq2		x		-1,95	-2,00	
gata transcription factor 9-like	comp22840_c0_seq1		x		-1,87	-1,71	
transcription factor glabra 3-like	comp38285_c0_seq1		x		-1,69	-1,42	
ap2-like ethylene-responsive transcription factor ant-li	comp34589_c2_seq1		x		-1,68	-1,04	
transcription factor bim1	comp34658_c0_seq1		x		-1,50	-1,24	
transcription factor tga1	comp32326_c0_seq1		x		0,95	1,20	
probable wrky transcription factor 40-like isoform 1	comp40603_c0_seq2		x		1,29	1,58	
ap2 erf domain-containing transcription factor	comp19984_c0_seq1		x		1,64	1,09	
transcription factor myb48	comp33951_c0_seq4		x		1,68	1,25	
wrky transcription	comp22770_c0_seq1		x		3,22	3,85	
transcription factor upbeat1-like	comp31380_c0_seq1			x	-6,88		-5,82
transcription factor myb39-like	comp22962_c1_seq1			x	-5,61		-4,06
AP2 domain TF	comp39358_c1_seq1			x	-4,05		-3,85
myb transcription factor 2	comp31766_c0_seq1			x	-3,54		-2,95
transcription factor bhlh63-like	comp29996_c0_seq2			x	-2,89		-2,33
myb transcription factor mixta-like 8 protein	comp33123_c0_seq2			x	-2,29		-2,04
basic helix-loop-helix	comp34010_c0_seq1			x	-2,21		-2,36
transcription factor bhlh30	comp28241_c0_seq1			x	-2,06		-1,63
basic leucine zipper transcription factor-like protein	comp36940_c1_seq1			x	-1,49		-1,37
transcription factor bhlh148	comp30658_c0_seq2			x	-1,24		-1,13
ap2 domain-containing transcription factor	comp41507_c1_seq1			x	-1,13		-1,28
myb transcription factor	comp35426_c0_seq1			x	1,97		2,00

		"Common pool"	"Control specific pool"	"Leaf specific pool"	Cont Leaf DtoA	Cont LR BtoC	Pi Leaf DtoA
**Others**							
isp4-like protein OpT4 oligo petide transporter	comp42126_c1_seq5	x			-7,77	-1,86	-7,06
rna recognition motif-containing protein	comp40659_c0_seq6	x			-6,39	-2,75	-6,63
tpx2 (targeting protein for xklp2) protein family	comp43768_c1_seq16	x			-6,02	-7,25	-6,19
mate efflux family protein chloroplastic-like	comp30797_c0_seq1	x			-4,09	-1,96	-3,52
harpin-induced family protein	comp30081_c0_seq1	x			-3,72	-2,20	-3,59
syntaxin-related protein knolle	comp31689_c0_seq1	x			-3,67	-2,76	-3,11
hmg (high mobility group) box protein	comp39171_c1_seq1	x			-3,29	-2,79	-2,51
leucine-rich repeat family protein	comp41131_c0_seq1	x			-3,21	-2,11	-2,95
p-loop containing nucleoside triphosphate hydrolase-li	comp41045_c0_seq1	x			-3,17	-2,12	-2,47
p-loop containing nucleoside triphosphate hydrolase-li	comp41837_c0_seq1	x			-3,14	-2,73	-2,00
protein iq-domain 1-like	comp41104_c0_seq1	x			-1,70	-1,31	-1,39
iq domain-containing protein	comp42249_c0_seq2	x			-1,63	-2,17	-1,64
quasimodo1-like protein	comp42130_c0_seq1	x			-1,17	-1,07	-1,07
calmodulin binding	comp32225_c0_seq1	x			-1,08	-1,01	-1,26
50s ribosomal protein l1	comp36790_c0_seq1	x			1,09	1,06	1,18
late embryogenesis abundant protein	comp33802_c0_seq1	x			1,43	3,24	1,34
multidrug resistance	comp42525_c0_seq1	x			1,69	2,12	2,00
protein prolifera	comp39887_c0_seq1	x			3,25	8,21	3,47
e3 ubiquitin-protein ligase xbat31-like	comp39201_c1_seq3	x			5,00	5,44	4,49
transducin wd-40 repeat-containing protein	comp43499_c0_seq2		x		-8,81	-6,68	
rna recognition motif-containing protein	comp40659_c0_seq1		x		-7,02	-3,00	
early nodulin-like protein 1-like	comp27613_c0_seq1		x		-4,81	-1,50	
branchless trichome protein	comp26646_c0_seq1		x		-4,68	-2,42	
cop1-interacting protein 7	comp32450_c0_seq1		x		-4,34	-2,92	
early nodulin-like protein	comp29078_c0_seq1		x		-3,82	-2,74	
f-box lrr-repeat protein 17-like	comp31563_c0_seq1		x		-3,78	-2,70	
dnaj heat shock n-terminal domain-containing protein	comp38770_c0_seq1		x		-3,37	-2,38	
protein trichome birefringence-like 36	comp29700_c0_seq1		x		-3,30	-2,45	
tpx2 (targeting protein for xklp2) family protein	comp22227_c0_seq2		x		-3,19	-1,54	
c2 domain-containing protein	comp40522_c0_seq1		x		-3,18	-2,26	
calmodulin binding	comp32855_c0_seq1		x		-3,08	-1,94	
p-loop containing nucleoside triphosphate hydrolase-li	comp38157_c0_seq1		x		-2,94	-2,77	
tpx2 (targeting protein for xklp2) family protein	comp39632_c0_seq1		x		-2,80	-2,91	
hva22-like protein c	comp44182_c0_seq1		x		-2,68	-2,43	
calmodulin-like protein	comp44074_c0_seq1		x		-2,44	-2,22	
transducin wd40 domain-containing protein	comp40265_c0_seq1		x		-2,38	-2,58	
blue copper protein	comp28458_c0_seq1		x		-2,27	-2,27	
protein iq-domain 31-like	comp20089_c0_seq1		x		-2,13	-1,55	
nodulin-like protein	comp40007_c0_seq1		x		-2,03	-1,32	
protein iq-domain 21	comp33322_c1_seq1		x		-1,75	-1,58	
trichome birefringence-like 27 protein	comp40064_c0_seq1		x		-1,54	-0,79	
cell elongation protein diminuto	comp43942_c0_seq1		x		-1,45	-1,81	
jumonji domain protein	comp38312_c0_seq1		x		-1,40	-1,18	
f-box kelch-repeat protein	comp38383_c0_seq1		x		-0,78	-1,08	
splicing factor 3b subunit 2	comp41061_c2_seq5		x		0,89	1,43	
calmodulin	comp20085_c0_seq1		x		1,07	0,72	
ring u-box domain-containing protein	comp39024_c0_seq1		x		1,24	0,89	
conserved hypothetical protein	comp31883_c0_seq1		x		1,85	2,87	
calcium-binding protein cml42	comp30734_c0_seq1		x		1,95	1,36	
sigma factor binding protein	comp20038_c0_seq1		x		2,15	1,91	
mate efflux family protein alf5-like	comp33238_c0_seq1		x		2,52	3,00	
sensitive to freezing 6 protein	comp42472_c0_seq4		x		2,56	3,16	
dna mismatch repair protein msh6-1	comp43777_c2_seq6		x		3,29	1,91	

		"Common pool"	"Control specific pool"	"Leaf specific pool"	Cont Leaf DtoA	Cont LR BtoC	Pi Leaf DtoA
transducin wd-40 repeat-containing protein	comp26403_c0_seq1			x	-5,97		-6,07
kelch repeat-containing f-box family protein	comp34217_c0_seq1			x	-5,48		-3,97
protein iq-domain 18	comp31535_c0_seq1			x	-5,15		-4,59
ddb1- and cul4-associated factor homolog 1-like	comp43378_c5_seq3			x	-4,05		-2,91
early nodulin	comp43521_c0_seq4			x	-2,84		-2,79
aquaporin	comp37244_c0_seq1			x	-2,05		-1,75
low temprature induced-like protein	comp40892_c1_seq4			x	-1,98		-1,76
rna recognition motif-containing protein	comp42946_c2_seq1			x	-1,84		-1,26
c2 domain-containing protein	comp39829_c0_seq1			x	-1,81		-1,44
protein iq-domain 1-like	comp32461_c0_seq1			x	-1,78		-1,98
hva22-like protein i	comp32026_c1_seq1			x	-1,51		-1,48
transducin wd40 domain-containing protein	comp36774_c0_seq2			x	-1,37		-1,22
harpin-induced protein	comp22936_c0_seq1			x	-1,34		-1,32
protein iq-domain 32-like	comp40764_c0_seq1			x	-1,20		-0,89
cop1-interacting protein	comp36999_c0_seq1			x	-1,13		-0,95
calmodulin-related protein	comp34974_c0_seq1			x	-1,05		-1,17
mate efflux family protein dtx1-like	comp35401_c0_seq1			x	0,91		0,83
50s ribosomal protein	comp28448_c0_seq1			x	0,92		1,03
chaperone protein dnaj	comp30687_c1_seq1			x	0,93		0,98
aarf domain-containing protein	comp31855_c0_seq1			x	0,97		0,88
clavaminate synthase-like protein	comp43386_c0_seq1			x	1,05		1,00
spx domain-containing protein	comp23243_c0_seq1			x	1,07		1,20
protein srg1	comp42139_c0_seq1			x	1,10		1,16
metal-nicotianamine transporter ysl3	comp41205_c0_seq1			x	1,22		1,21
kelch repeat-containing protein	comp37566_c0_seq1			x	1,58		2,01
nbs-lrr resistance protein	comp43296_c1_seq1			x	2,09		1,89
kelch repeat-containing protein	comp37566_c0_seq2			x	2,41		2,29
metal-nicotianamine transporter ysl1	comp37162_c0_seq1			x	2,48		1,47
nudix hydrolase 2-like AtNUDT7	comp43278_c0_seq10			x	2,85		3,57
transparent testa 12 (MATE efflux family protein 7-like)	comp43568_c0_seq5			x	3,76		6,13
